# *Drosophila Appl* loss disrupts proteins in mitochondrial oxidative and glycosylation pathways conserved in Alzheimer's disease

**DOI:** 10.1016/j.isci.2026.117781

**Published:** 2026-10-12

**Authors:** Zachary M. Smith, Fernando J. Vonhoff

**Affiliations:** 1 Department of Biological Sciences, University of Maryland, Baltimore County, Baltimore, MD, USA

**Keywords:** amyloid precursor protein, APP, genome assembly, proteomics, glycosylation, mitochondrial metabolism, lipid oxidation, CRISPR mutant, Appl^d^, *Drosophila melanogaster*

## Abstract

The physiological functions of the amyloid precursor protein (APP) remain unclear due to genetic redundancy and complex phenotypes. For decades, *Drosophila* amyloid precursor protein-like (*Appl*) research has relied on *Appl*^d^, a presumed null allele generated by irradiation. Here, we show that *Appl*^d^ contains complex chromosomal rearrangements and background mutations confounding the interpretation of gene function. To address this, we created a precise CRISPR toolkit, including a clean null and a reporter allele. Deep proteomic profiling reveals that true *Appl* loss affects proteins associated with N-linked glycosylation, mitochondrial, and metabolic functions in a distinct signature from the background of *Appl*^d^. This conserved metabolic signature mirrors alterations in human patients and mouse Alzheimer's models and is associated with disrupted sleep and locomotion in flies. Our findings correct the genetic understanding of *Appl*, establishing it as a conserved regulator of metabolic balance essential for neuronal function.

## Highlights

• Long-read sequencing reveals legacy *Appl* allele is a complex rearrangement
• CRISPR-engineered Appl alleles enable precise genetic analysis
• Multi-platform proteomics separates background effects from shared Appl signature
• *Appl* loss signature is conserved across mouse models and human Alzheimer disease

## Graphical abstract

**Figure:**
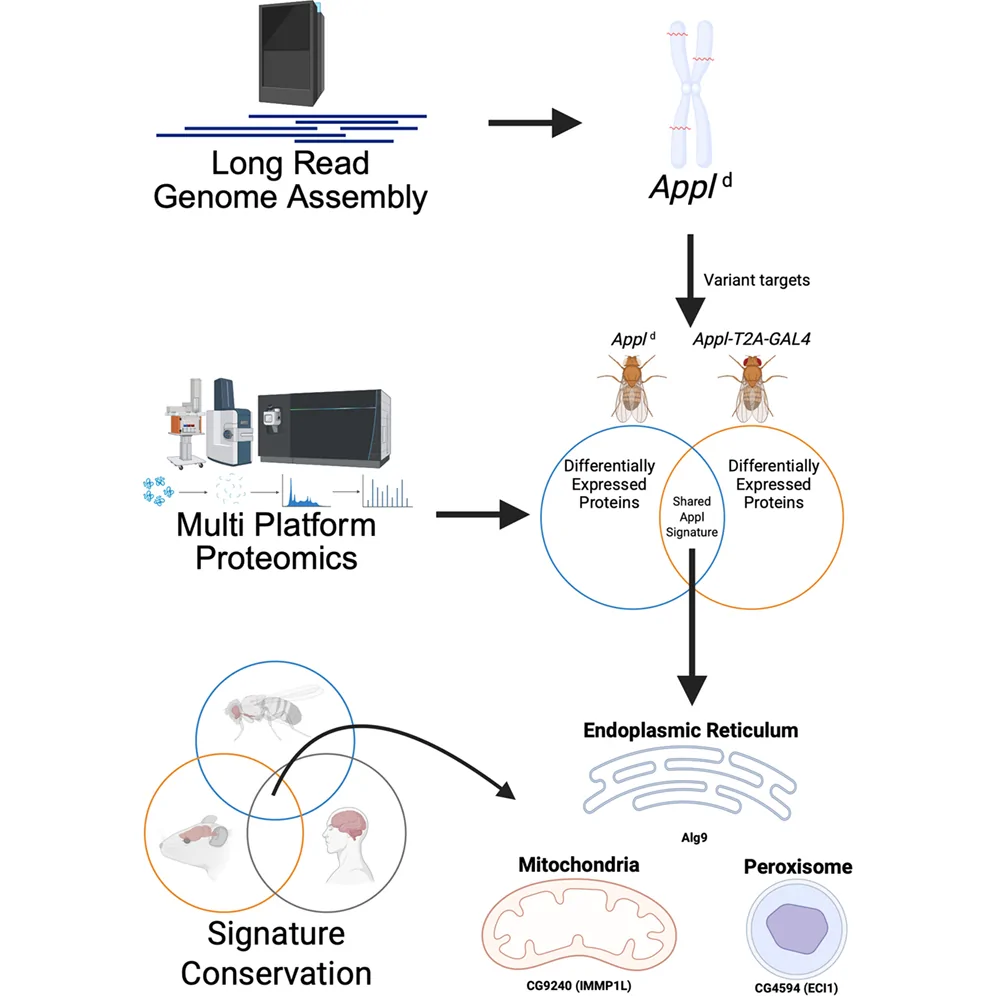

## Teaser

Human metabolism; Genetics; Neuroscience

## Introduction

The human amyloid precursor protein (APP) is a large, single-transmembrane protein whose function is regulated by proteolytic processing, yielding various fragments with distinct cellular roles.^1^^,^^2^ One such fragment, amyloid-beta (Aβ), generated by sequential processing of APP by the β- and γ-secretase complexes, is a hallmark of Alzheimer's disease (AD).^3^ Despite its well-established connection to AD, the normal physiological functions of APP remain unclear, even after decades of detailed studies of its individual domains.^3^ Understanding APP’s physiological roles that are independent of amyloid pathology is increasingly recognized as essential for deciphering how normal synaptic and metabolic functions shift toward degeneration in AD.

Because the essential functions of APP have been obscured by redundancy and embryonic lethality in vertebrates, researchers have turned to other model systems.^4^^,^^5^^,^^6^ *Drosophila melanogaster*, which expresses a single APP-family ortholog, the amyloid precursor protein-like (Appl), provides a genetically powerful system to study the conserved signaling and trafficking mechanisms regulated by APP family proteins *in vivo.*^7^ Unlike mammals, which express three partially redundant APP-family proteins, *Drosophila* express a single ortholog, Appl, simplifying loss-of-function analysis while preserving conserved trafficking and signaling features of the APP family.^8^^,^^9^^,^^10^ This makes *Drosophila* particularly useful for identifying core APP-family functions that may be obscured by redundancy among mammalian isoforms. Appl has been implicated in synapse formation, neuronal survival, and behavioral regulation, and for over three decades, these insights have relied on a single presumed null allele, *Appl*^d^.^8^^,^^10^^,^^11^^,^^12^^,^^13^ Despite its central role, the genetic architecture of *Appl*^d^ remains incompletely characterized.

*Appl*^d^ was generated using a high-dose irradiation strategy that induced translocations from chromosomes carrying *Appl* deficiencies, ultimately identifying a single male with the desired X chromosome translocation among approximately 6,000 progeny.^8^ Given that even low-dose irradiation (∼1 Gy, equivalent to ∼100 rad) dramatically increases germline mutation frequency in *Drosophila*, the 4,000 rad (∼40 Gy) dose used to generate *Appl*^d^ was approximately 40-fold higher and likely introduced additional background mutations.^8^^,^^14^ Although the allele was transformative for early research and enabled functional insights into Appl’s role, this mutagenesis approach, along with limited characterization of the *Appl* locus, raises the possibility that *Appl*^d^ contains both structural rearrangements and off-target variants that could confound previous findings. Given the reliance of nearly all functional Appl studies on this allele, unrecognized background mutations could have shaped the conclusions about Appl biology.

With the advent of long-read sequencing and CRISPR genome engineering, it is now possible to directly address these limitations of *Appl*^d^. Resolving these uncertainties is crucial not only for the *Drosophila* field but also for understanding conserved APP mechanisms relevant to human neurodegenerative diseases. Here, we combine *de novo* long-read genome assembly with quantitative proteomics to define the molecular structure of *Appl*^d^ and establish a toolkit of precisely CRISPR-engineered *Appl* mutants, including a null driver (*Appl*-T2A-GAL4) and an endogenously C-terminal GFP-tagged line. Direct comparison of proteomic signatures from *Appl*^d^ and *Appl*-T2A-GAL4 reveals core pathways regulated by Appl, such as glycosylation, protein trafficking, and mitochondrial metabolism, and also contextualizes decades of research conducted in the *Appl*^d^ background. Together, these results provide a high-resolution molecular view of APP loss-of-function effects and establish a genetically precise foundation for studying conserved APP family pathways relevant to synaptic maintenance and AD.

## Results

### High-quality genome assembly of *Appl*^d^ provides basis for *Appl* locus characterization

Because nearly all functional work on Appl depends on the *Appl*^d^ line, defining its genomic structure was the crucial first step toward understanding its molecular composition. To identify large indels and structural variants in *Appl*^*d*^, especially on the X chromosome, we performed long-read genome sequencing using the PacBio Revio system. This generated 333,998 circular consensus HiFi reads (mean length = 10.4 kb, minimum quality = 0.999; Figure 1A).^15^ GenomeScope 2.0 modeling of the 21-mer spectra indicated a haploid genome size of 136.6 Mb, with ∼76% unique sequence and ∼32.7 Mb repetitive content (model fit = 98.5%).^16^ The modeled k-mer peak coverage (∼12×) was consistent with the low heterozygosity expected in laboratory *Drosophila* strains. Mapping of the reads to the *Drosophila melanogaster* reference genome (dm6, r6.62) with minimap2 yielded uniform coverage (mean depth = 23.7×; Figure 1B), with the expected ∼50% drop on the X chromosome confirming a male-derived sample.^17^^,^^18^^,^^19^

**Figure 1:**
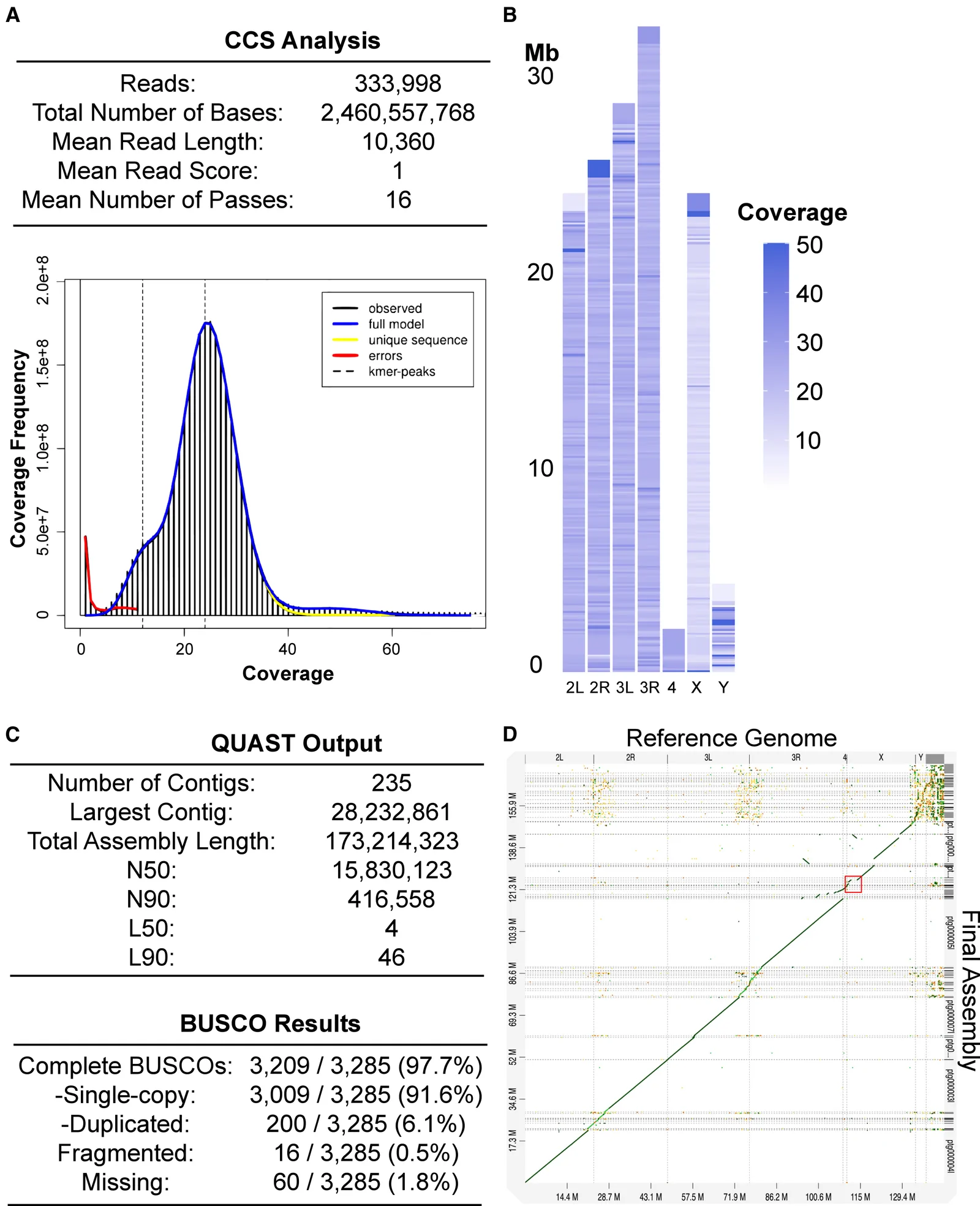
Genome sequencing, assembly, and quality assessment of *Appl*^d^*Drosophila* line (A) Circular consensus sequencing (CCS) metrics for the PacBio Revio. The run generated 333,998 HiFi reads, totaling 2.46 Gb, with an average read length of 10.4 kb and a quality score of 0.999 or higher. K-mer frequency (k = 21) distribution was calculated using Jellyfish and modeled with GenomeScope 2.0, revealing a primary k-mer peak (kcov) of 12×, which indicates low heterozygosity and high-quality reads. The genome was estimated to be 136.6 Mb, with 76% being unique sequences. (B) Chromosome heatmap of reads aligned in 100 kb bins to the reference (r. dm6.62) using minimap2 and visualized in RStudio. Read depth is evenly distributed across autosomes and decreases by half on the X chromosome, confirming the data were collected from male individuals. (C) QUAST analysis of the final Racon- and Merfin-polished assembly shows 235 contigs totaling 173.2 Mb, with an N50 of 15.8 Mb and an L50 of 4. BUSCO analysis using the Diptera (odb10) lineage as a reference indicates high completeness (97.7%) and high single-copy percentage (91.6%). (D) D-GENIES plot of alignment between *Appl*^d^ assembly (*y* axis) and reference genome (r. dm6.62; *x* axis) displays high conservation along the chromosomal arms, with quality declining near the centromeric and telomeric regions. Alignment quality is shown by color where green indicates high identity matches (>95%), and yellow and orange indicate lower identity alignment. The *Appl* region is highlighted within the red box.

*De novo* assembly with hifiasm (v.0.24) produced a 173.2 Mb primary assembly comprised of 235 contigs with high contiguity and completeness (N50 = 15.8 Mb; BUSCO = 97.7%) (Figure 1C).^20^^,^^21^^,^^22^ Whole-genome alignment with D-GENIES revealed near-perfect synteny across chromosome arms, with only minor fragmentation at centromeric and telomeric regions (Figure 1D).^23^ In D-GENIES plots, strong green diagonals represent high identity alignments (>95%), while yellow and orange colorations indicate lower-quality alignments, often in repetitive or fragmented regions. These results demonstrate that our HiFi dataset enables assembly close to reference quality, offering a reliable foundation for downstream analysis of structural variants and locus reconstruction. Using this high-quality assembly, we aimed to detect structural and sequence differences between *Appl*^d^ and the reference genome. The high-fidelity assembly facilitated the identification of structural disruptions that directly affect the *Appl* locus.

### Long-read assembly reveals fragmentation of the *Appl* locus in *Appl*^d^

Analysis of the assembled genome revealed that the *Appl* locus is fragmented by two large deletions, redefining the long-assumed structure of *Appl*^d^. To assess genomic variation in the *Appl*^d^ mutant relative to the reference genome, we applied two complementary variant callers: FreeBayes, a long-indel-sensitive variant caller, and Longshot, a HiFi-optimized caller.^24^^,^^25^ FreeBayes identified 837,318 variants, and Longshot detected 782,501 SNPs, with a majority of the sites being shared calls (554,130; 54.3%) (Figure 2A). Variants were distributed across all chromosome arms with a similar occurrence (Figure 2B).

**Figure 2:**
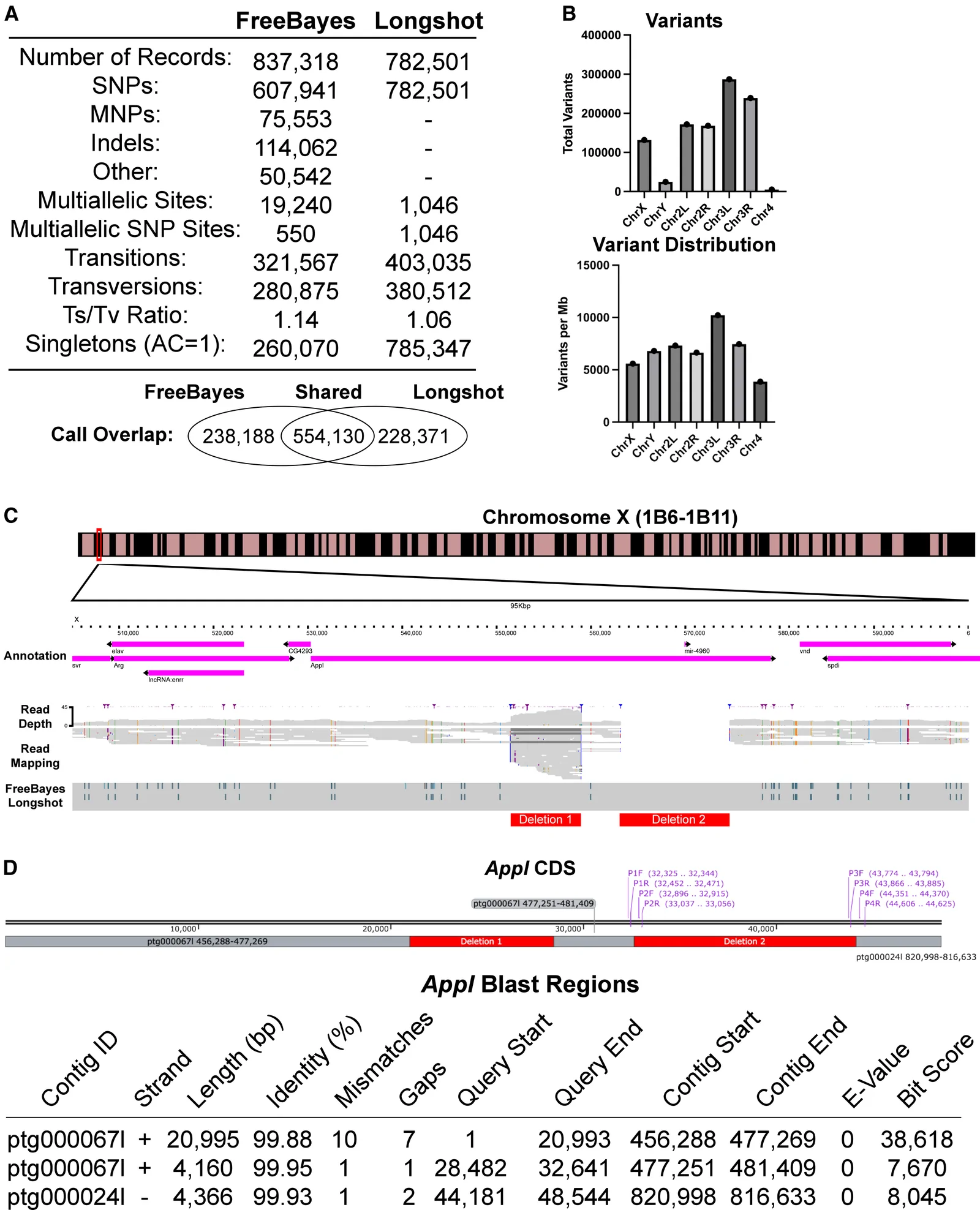
Structural and variant analysis of the *Appl* locus in the *Appl*^d^*Drosophila* genome (A) Summary of variant-calling statistics from both FreeBayes and Longshot. Highlighted similarities and differences in the callers with 54.3% being shared calls. (B) Variant calls calculated and visualized per chromosome arm and frequency of variants per length. (C) Mapped locus of the X chromosome (UCSC) with the reference genome visualized (r. dm6.62) in JBrowse 2, showing gene annotations in magenta, followed by read depth and individual reads, with variants called at the bottom. (D) BLAST alignment of *Appl* CDS against the assembly to show three major fragments and two major deletions. Purple annotations correspond to primers used in Nithianandam et al.^26^ Table showing exact blast coordinates and hits of contigs.

We next focused on structural variation within the *Appl* locus. Read mapping revealed two distinct gaps in read coverage. The first, designated as Deletion 1 (∼7 kb), corresponds to a highly repetitive sequence. Although individual reads mapped within this region, no single read spanned both upstream and downstream regions, indicating a loss of this segment within the *Appl* locus (Figure 2C). The second gap, Deletion 2 (∼11.5 kb), showed a complete absence of mapped reads, consistent with a second deleted region within the locus (Figure 2C). Together, these mapping data support *Appl* locus fragmentation into three regions by two deletions in *Appl*^d^.

To investigate the genomic context of these fragments, we performed BLASTn searches against our *de novo* assembly (Figure 2D).^27^ Two contigs contained all non-repetitive *Appl* regions: ptg000067l, which contains the 5′ region and the sequence between Deletion 1 and Deletion 2, and ptg000024l, which contains the 3′ region downstream of Deletion 2. Visualization of BLASTn hits across the locus confirmed the fragmentation and supported the presence of both deletions (Figure S1). Importantly, the boundaries of these deletions are consistent with the irradiation-induced deficiency described in Luo et al. (1992) and with the primer walking results of Nithianandam et al. (2023) (Figure 2D).^8^^,^^26^

To better place these fragments in a broader genomic context, we next visualized the BLASTn hits alongside gene annotations transferred from the reference genome using Liftoff.^27^^,^^28^ BLASTn revealed that the 5′ contig (ptg000067l) contains *elav* upstream of *Appl*, while the 3′ contig (ptg000024l, reverse complemented) contains the 3′ fragment of *Appl* followed by *vnd* (Figures 3A and 3B). Liftoff annotation confirmed that these flanking genes are syntenic with the reference genome and that intervening genes also matched their expected order and orientation (Figure 3B). This concordance between local BLAST hits and transferred gene models supports the reconstruction of a contiguous pseudo-locus (PseudoX) by concatenating ptg000067l and the reverse complement of ptg000024l, with a 20-nucleotide artificial spacer inserted between them. This spacer does not represent genomic sequence but is included to clearly mark the unresolved junction. No reads span the junction between these contigs in our dataset, and testing all eight possible contig permutations similarly yielded no spanning reads (Supplemental File 25). Clipped read analysis also failed to identify a junction, instead mapping to sequences from other chromosomes.

**Figure 3:**
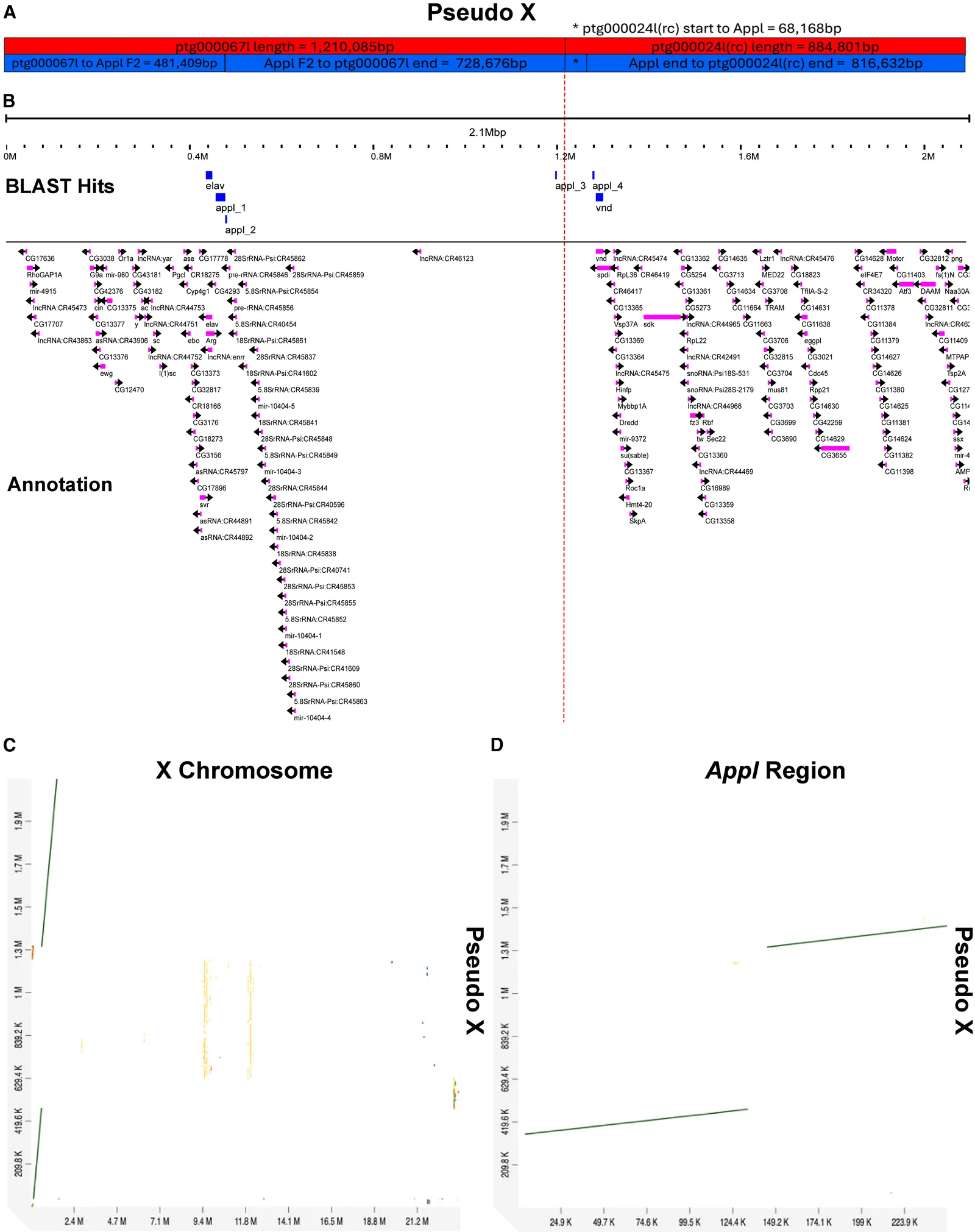
Reconstruction and structural mapping of the *Appl* locus (A) Schematic representation of reconstructed region (PseudoX) created by joining ptg000067l and the reverse complement of ptg000024l (red boxes) from the final assembly. The Appl CDS is fragmented across multiple regions (blue boxes). (B) BLAST confirmed the location of both nearby genes and *Appl* fragments shown in blue. Hits are shown for *Appl* labeled 1–4, with hit 3 being a likely off-target blast hit. Liftoff annotation of genes from the reference genome is shown in magenta. Contig end delineated by a vertical red hashed bar. (C) Dot plot of PseudoX (*y* axis) against the reference X chromosome (*x* axis) showing apparent synteny with a large insertion gap. Alignment quality indicated by color where green indicates high identity matches (>95%), and yellow and orange indicate lower alignment. Yellow dotted region likely indicates a highly repetitive region. (D) Alignment of the same PseudoX (*y* axis) with the *Appl* locus (±100 kb) (*x* axis) showing clear insertion.

Additionally, these *Appl* regions are not adjacent to each other at the ends of each contig; instead, the *Appl* fragments are located near the middle of each contig, suggesting the possible insertion of non-native DNA. Together, these results indicate unresolved structural complexity near the *Appl* locus that will require additional sequencing or supplementary scaffolding approaches to fully resolve. However, based on synteny, our PseudoX reconstruction provides the best available prediction of contig orientation around the *Appl* locus, as supported by the D-GENIES alignment to the reference genome (Figures 3C and 3D). Although these analyses define the precise deletions and genomic fragmentation in *Appl*^d^, the extensive structural complexity and background variation in this fly line limit its utility for dissecting Appl-specific functions. This motivated us to generate precisely engineered mutants to enable targeted investigation of Appl-associated molecular pathways.

### CRISPR-based *Appl* mutants enable precise dissection of Appl function

To overcome the limitations of the current model, *Appl*^d^, we generated a suite of CRISPR-engineered *Appl* mutants designed for precise functional dissection (Figure 4). These mutants include an endogenously C-terminal GFP-tagged allele, which enables detection and antibody-based localization of Appl, and a null-driver T2A-GAL4 in which a large portion of the *Appl* coding sequence is deleted and replaced by a T2A-GAL4 cassette in frame with the remaining coding exons, enabling cell-specific rescue and manipulation (Figure 4; Figure S2).^29^ Both alleles were validated at the genomic level by Sanger sequencing, and protein expression was documented using western blotting. *Appl*-GFP protein expression was quantified relative to endogenous Appl, and confocal imaging shows GFP signal in the early (L1) and late (L3) larval nervous system (Figures S2A–S2C). Because Appl-GFP expresses approximately 10% of endogenous Appl levels, endogenous GFP fluorescence is dim, and this allele is best suited to detection and antibody-based imaging rather than direct visualization of fluorescence. Co-labeling with ATP5A provides organelle-level context for *Appl*-GFP localization in the late larval brain and ventral nerve cord (VNC) (Figure S2C). Additionally, *Appl*-T2A-GAL4 null was confirmed as a protein-null allele using western blot and robustly drove UAS-CD4::tdTomato expression in both the larval CNS and adult brain (Figure 4; Figure S2D). Together, these fly lines provide the tools needed to conduct proteomic profiling of both the null mutant and the legacy *Appl*^d^, with the goal of identifying molecular changes associated with *Appl* loss. Overall, these fly lines reduce the confounding background effects inherent to *Appl*^d^ and provide a genetically defined framework for analyzing Appl biology with cell type and temporal precision.

**Figure 4:**
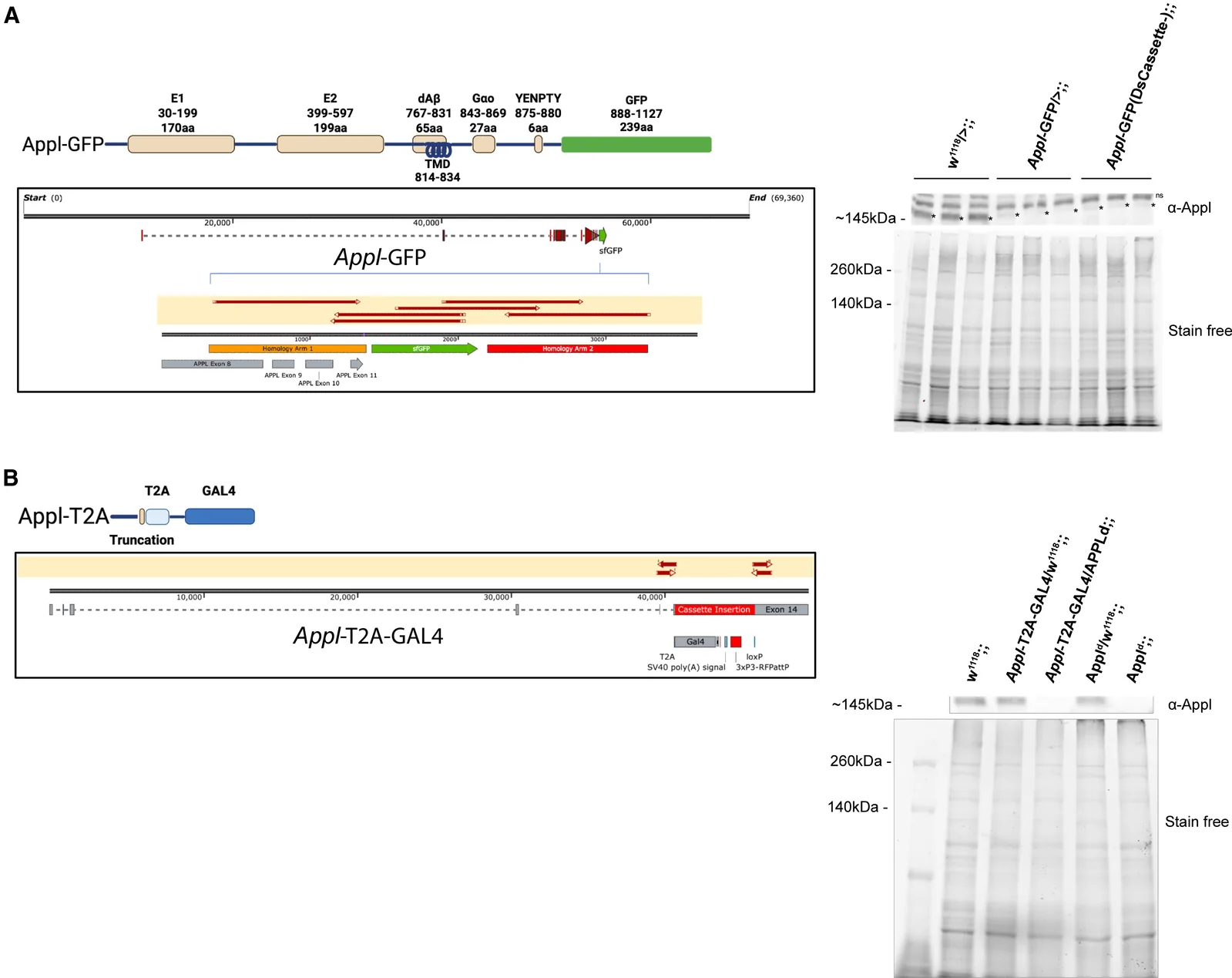
CRISPR-generated Appl mutants and validation (A) Genomic integration schematic of the C-terminal *Appl*-GFP allele (top), generated by inserting a GFP tag at the end of the endogenous Appl locus. Gene region and sequencing (maroon arrows) confirming the GFP junctions (bottom). Western blot of adult head lysates (male 3–5 days old) (40 μg/lane) shows full-length Appl in *w*^1118^, *Appl*-GFP with DsRed cassette, and *Appl*-GFP after DsRed excision (right). Lysates prepared using RIPA (Thermo Fisher Scientific) with protease inhibitor (Pierce), quantified using BCA (Pierce), resolved on 7.5% TGX stain-free gels (BioRad), transferred to PVDF, and probed overnight with pre-adsorbed chicken anti-Appl (Philip Copenhaver) followed by rabbit anti-chicken (Proteintech). Asterisks (∗) to the right of bands denote the specific Appl signal used for quantification; “ns” to the right of the blot denotes the row of nonspecific bands. Quantification of protein expression is shown in Figure S2A; *Appl*-GFP images are shown in Figures S2B and S2C. (B) Genomic schematic of the *Appl*-T2A-GAL4 allele (top), with schematic and Sanger sequencing to confirm junctions (bottom). Western blot was run using the same conditions as the *Appl*-GFP blot in (A), using lysates from 3- to 5-day-old female heads, confirming the loss of Appl in *Appl*-T2A-GAL4 flies (right).

### Proteomic profiling defines core Appl pathways and contextualizes *Appl*^d^ background effects

To determine how loss of Appl affects the neuronal proteome and to disentangle true Appl-dependent changes from *Appl*^d^ background effects, we performed complementary Orbitrap and timsTOF analysis. Together, these datasets revealed extensive proteomic changes in *Appl*^d^. We observed significant enrichment in oxidative metabolism (Figure 5A; Figure S3), alongside dysregulation of RNA processing and ribosomal pathways, which indicates disrupted proteostasis. This signature is distinct from *Appl*-T2A-GAL4 (Figure 5B). While pathway analysis revealed distinct signatures relative to their controls (Figures 5A and 5B), direct comparisons between the lines highlight background-dependent effects (Figure 5C). Given this divergence, we sought to better define a high-confidence *Appl* loss proteomic signature.

**Figure 5:**
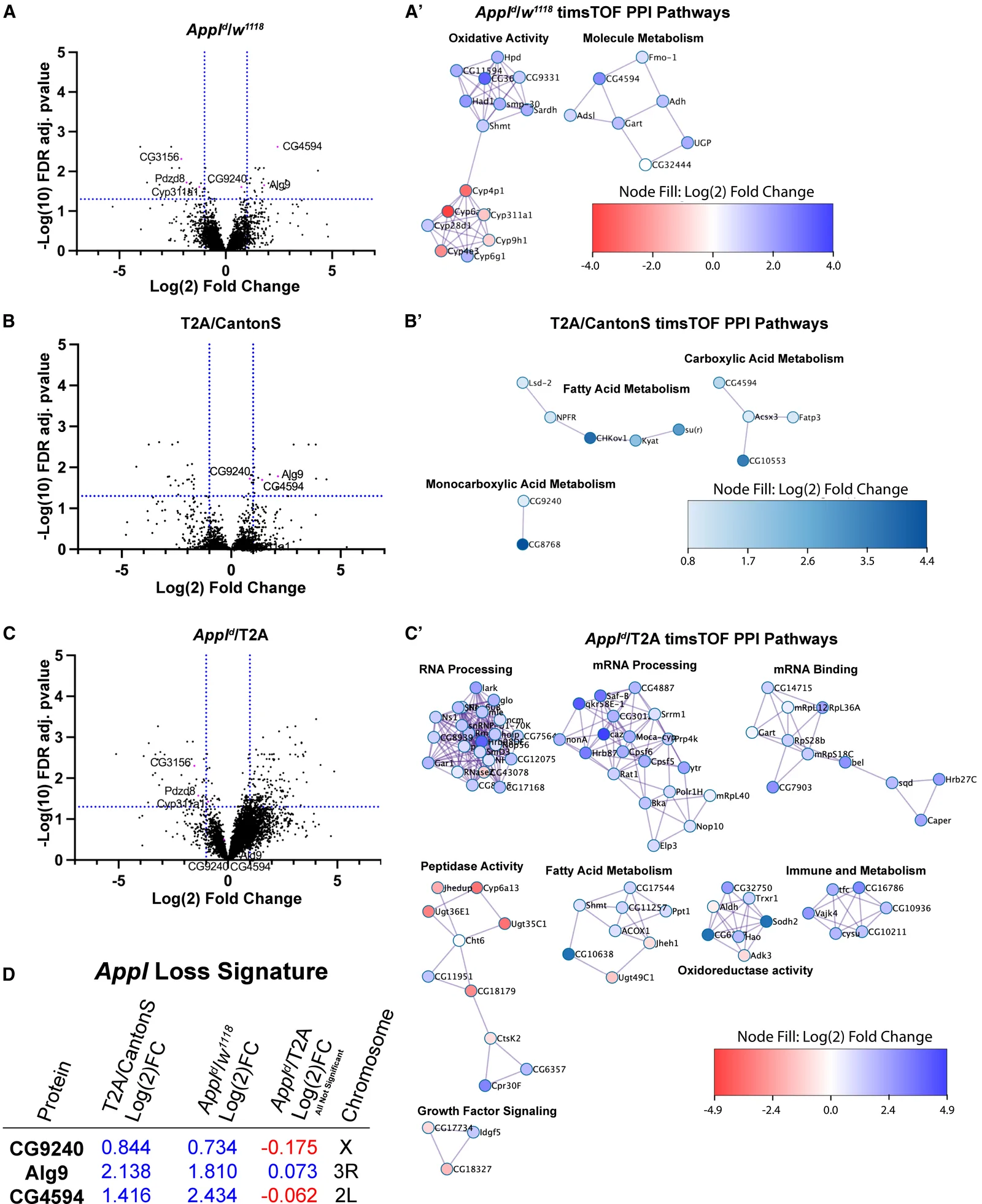
Proteomic analysis reveals Appl-dependent proteomic dysfunction Proteomic profiling was employed to define the core pathways associated with Appl, distinguishing Appl-dependent changes from background effects inherent to *Appl*^d^. (A–C) Volcano plots showing Log2(FC) (*x* axis) versus –Log10(adj. *p* value) (*y* axis) for proteins quantified in each comparison: (A) *Appl*^d^ vs. *w*^1118^, (B) *Appl*-T2A-GAL4 vs. Canton-S, and (C) *Appl*^d^ vs. *Appl*-T2A-GAL4. Blue vertical lines indicate ±1 of Log2(FC), and the horizontal blue line indicates a transformed significance threshold corresponding to an adj. *p* value of 0.05. Selected proteins were identified to be Appl or *Appl*^d^ Signature through filtering. (A′-C′) Pathways correspond to Metascape protein-protein interaction maps, where nodes are colored by Log2(FC) (blue is upregulated; red is downregulated in specific comparisons). Clusters are annotated by using generalized GO terms produced for each pathway. Specific pathway targets are available in the supplemental material (Supplemental Folder 6). (D) Table summarizing expression of three candidate proteins fitting Appl-specific filtering criteria (significant in both *Appl*^d^/*w*^1118^ and *Appl*-T2A-GAL4/Canton-S but not significant in *Appl*^d^/*Appl*-T2A-GAL4).

To define a high-confidence *Appl* loss proteomic signature, we filtered timsTOF results for proteins significantly altered in both *Appl*^d^ to *w*^1118^ and *Appl*-T2A-GAL4 to Canton-S comparisons but not significantly changed in *Appl*^d^ to *Appl*-T2A-GAL4. This resulted in a set of three proteins: CG9240, Alg9, and CG4594 (Figure 5D). Two of which (Alg9 and CG4594) were also significant in the Orbitrap dataset (Figure S3). Alg9 is a mannosyltransferase required for N-linked glycosylation, a modification that is essential for proper protein folding and trafficking, while *CG4594* encodes a predicted mitochondrial isomerase implicated in fatty acid oxidation and amyloid-related metabolic dysfunction.^30^^,^^31^^,^^32^^,^^33^ Consistent with these changes, inspection of our Orbitrap dataset for proteins carrying post-translational modifications revealed multiple cytoskeletal regulators with altered phosphorylation levels (Figure S4). These conserved hits focus on glycosylation and mitochondrial metabolism, two processes disrupted in APP-related neurodegeneration, indicating a common molecular pathway between fly and human APP biology.^34^^,^^35^^,^^36^^,^^37^

To identify *Appl*^d^-specific background effects, we filtered for proteins that significantly differ between *Appl*^d^ and *Appl*-T2A-GAL4, and between *Appl*^d^ and *w*^1118^, but are not significant in Canton-S to *w*^1118^ comparisons (Figure 6A). This yielded a list of proteins potentially indicating background changes in *Appl*^d^, including five hits on the X chromosome (Figure 6B). Notably, three of these (Cyp311a1, Pdzd8, and CG3156) are downregulated and could be candidates for direct genomic disruption or pathway interference. All three of these candidates have also significantly changed in the same direction and to a similar magnitude in our Orbitrap dataset. While we did not detect alterations in the Cyp311a1 coding region, genomic analysis identified clear disruptions in the other two candidates. *CG3156* lies near the *Appl* locus and harbors a predicted frameshift truncation in our *Appl*^d^ genomic data, whereas *Pdzd8* harbors a Val21Glu missense mutation, potentially disrupting protein stability or folding (Figure 6C; Figure S5). Notably, mutations within transmembrane domains are disproportionately associated with protein mislocalization, suggesting this variant may directly impair Pdzd8 function.^38^ These proteins play key roles in essential processes, as the mammalian ortholog of CG3156 (ABCB10) is a mitochondrial transporter that stabilizes mitoferrin, enabling iron delivery, with its loss disrupting iron utilization and increasing oxidative stress signaling.^39^ Additionally, Pdzd8 is essential for synaptic bouton formation, regulates autophagy, and shows behavioral phenotypes.^40^^,^^41^^,^^42^ All of these processes have been noted in *Appl*^d^ studies, suggesting that Pdzd8 loss could contribute directly to reported synaptic and behavioral phenotypes in this fly line.^11^^,^^13^^,^^26^ To confirm these disruptions we identified in *Pdzd8* and *CG3156* are unique to *Appl*^d^, we sequenced the corresponding regions in *Appl*^d^, *Appl*-T2A-GAL4, Canton-S, and *w*^*1118*^ flies using Sanger sequencing. All lines except *Appl*^d^ retained intact sequence, confirming that these alterations are specific to *Appl*^d^ (Figure S6). This further validates that the assembly is correct and that the *Appl*^d^ proteomic signature reflects irradiation-induced background effects in addition to *Appl* loss.

**Figure 6:**
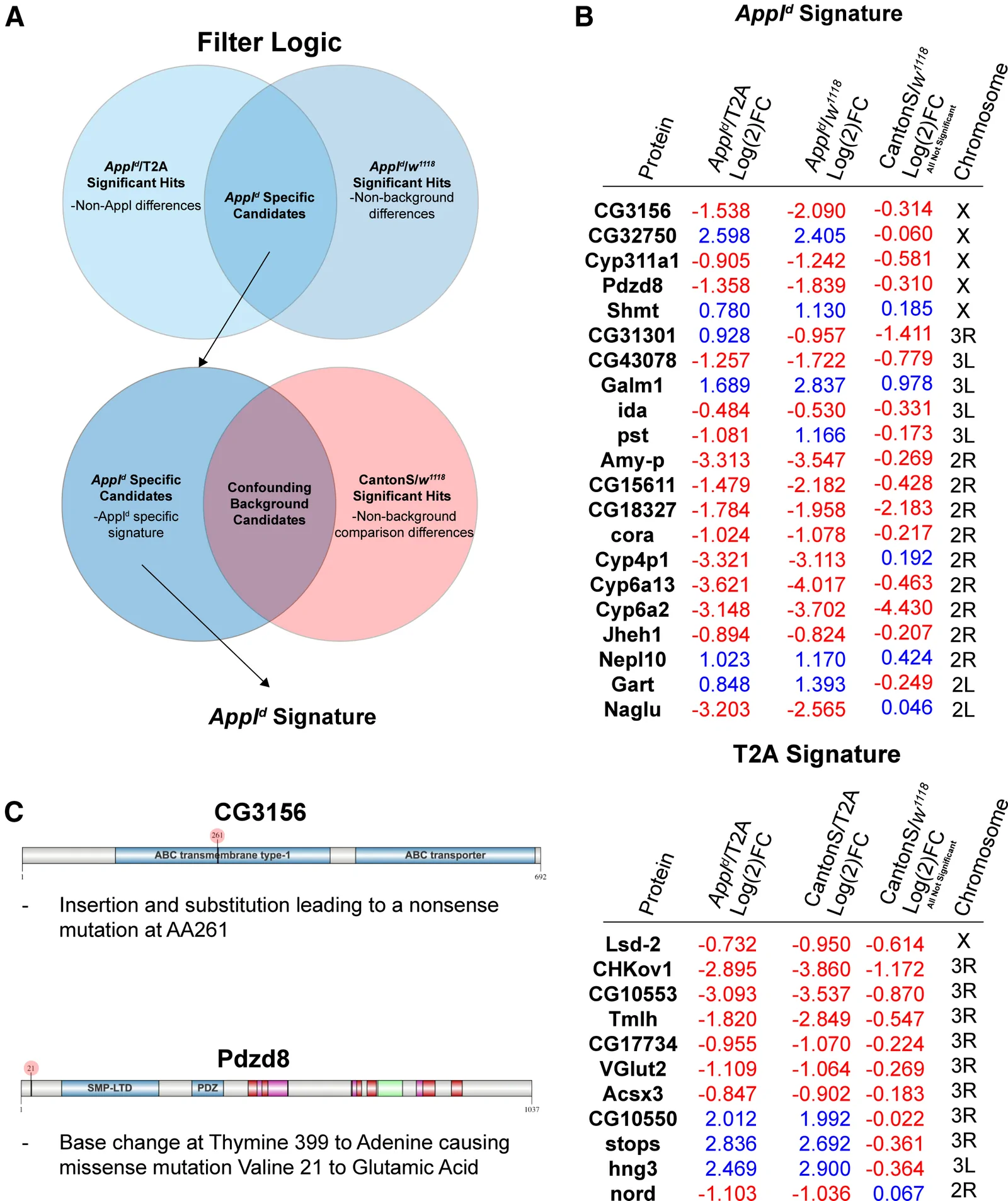
Identification of *Appl*^d^-associated proteomic changes and candidate genomic variants Comparison with the legacy *Appl*^d^ line uncovers specific background mutations and proteomic changes unrelated to *Appl* loss, contextualizing prior findings in *Appl*^d^. (A) Workflow used to define the *Appl*^d^ proteomic signature. Beginning with all detected proteins, we retained hits significant in both the *Appl*^d^ to *Appl*-T2A-GAL4 comparison and *Appl*^d^ to *w*^1118^ comparison to capture the *Appl*^d^ signature set of proteins. We then removed proteins significantly altered between Canton-S and *w*^1118^ to remove effects from the backgrounds of each strain. (B) Heatmap table of proteins found to be the *Appl*^d^ signature after filtering. Values represent Log2(FC) in all comparisons. Three select proteins are of interest due to their location on the X chromosome and are downregulated (CG3156, Pdzd8, and Cyp311a1) and additionally supported in both significance and change direction by the Orbitrap data (Figure S3 and Supplemental Folder 8). *Appl*-T2A-GAL4 signature was generated using similar filtering logic. (C) Schematic of proteins found to have predicted disruptive X-linked variants. *CG3156* harbors a two-base pair insertion and two subsequent substitutions (thymine and guanine insert after adenine 1188 [of CDS] and cytosine 1189 to adenine and adenine 1190 to thymine), resulting in a frameshift and premature termination codon at AA261. Pdzd8 contains a thymine-to-adenine substitution at nucleotide 399, causing a valine to glutamic acid missense mutation at position 21. Variants are visualized using IBS 2.0. Domains are represented in blue; disordered regions in red; phorbol ester/DAG-type in green; and basic, acidic, or polar residues in pink. Variations are observations that have not yet been determined to be causal. Additional protein sequence information is available in the supplemental data.

These findings suggest that irradiation-induced rearrangements in *Appl*^d^ disrupt additional X-linked genes, potentially confounding prior interpretations of *Appl*^d^ phenotypes. For consistency with earlier studies, we compared our *Appl*^d^ Orbitrap dataset with the dataset from Nithianandam et al., (2023) obtained from approximately 10-day-old *Appl*^d^ flies.^26^ This analysis showed partial overlap in protein expression patterns, as well as proteins with opposite changes, consistent with an additional age-related proteomic signature in *Appl*^d^ (Figure S7, Supplemental Folder 9). Although these overlaps were not part of the core Appl-specific profile defined here, they provide temporal context for interpreting *Appl*^d^ datasets and may help clarify age-related protein changes, both specific to this model and associated with aging overall.^43^

Because many of the dysregulated proteins in our Appl signature and *Appl*^d^ background participate in neuronal metabolism, trafficking, and synaptic maintenance—pathways disturbed early in AD—we next asked whether these molecular changes manifest as measurable behavioral phenotypes relevant to APP family biology. Prior studies of Appl have reported its roles in sleep architecture and locomotion, suggesting Appl molecular pathways may impact behavior.^44^^,^^45^ To test this directly in these mutants, we performed behavioral assays assessing rhythmic activity and motor performance.

### *Appl* mutants exhibit impaired locomotion and sleep dysregulation

Given that several Appl-dependent pathways regulate mitochondrial and glycosylation function, we next asked whether these disruptions could manifest as behavioral phenotypes. Using the widely established Drosophila Activity Monitor (Trikinetics), we tracked the activity of our Appl mutants to investigate the effects of *Appl* loss. Recent work demonstrated that cleavage of Appl and production of the intracellular domain (AICD) alter core clock gene expression and that nuclear AICD abundance differs across the day-night cycle.^44^ Although that study did not directly measure anticipation behavior, impairment of these proteolytic processes may affect Appl signaling and disrupt transitions around lights-on and lights-off. Sleep traces revealed that both *Appl*-T2A-GAL4 and *Appl*^d^ flies show broad alterations, including reduced latency (Figure 7A; Figure S8), and thus we focused our analysis specifically on these phase transitions. Additionally, pan neuronal Appl knockdown similarly leads to changes in various sleep parameters, including time spent sleeping, mean bout length, and the increase in sleep bout number during the night observed in *Appl*-T2A-GAL4 flies (Figure S8). Employing a custom R pipeline, adapted from previous analysis in Guerrero et al. (2025), we calculated both a morning (MAI) and an evening (EAI) anticipation index from activity bins before lights-on and lights-off, respectively.^46^ *Appl*^d^ exhibited a significantly higher MAI than *w*^1118^ controls (Figure 7B), whereas a significant decrease in MAI was observed in *Appl*-RNAi flies, similar to the slight, but not significant decrease in MAI observed in *Appl*-T2A-GAL4 mutants, when compared to controls. Consistently, T2A mutants displayed significantly decreased EAI relative to CantonS controls, which was partially recapitulated in *Appl*-RNAi flies, while *Appl*^d^ showed increased EAI compared to w^1118^ (Figure 7C). These results suggest *Appl* contributes to maintaining stable rest-activity transitions consistent with the previous AICD observations.^44^

**Figure 7:**
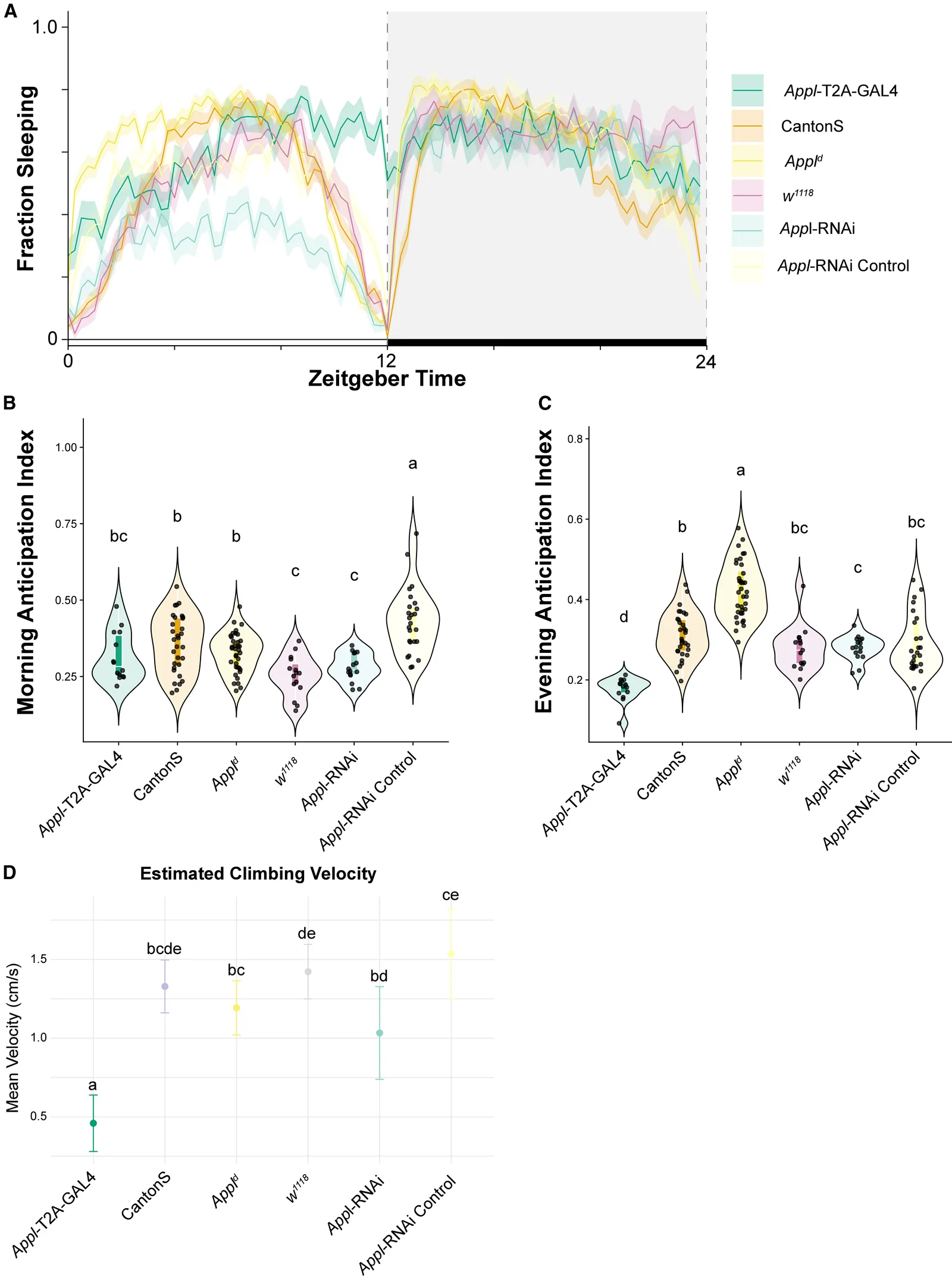
*Appl* loss disrupts circadian anticipation and locomotor performance (A) Sleep traces showing fraction of time asleep (mean ± SEM) across the 24 h light dark cycle. Data were analyzed in Vanessa-DAM and visualized in RStudio. White = light; gray = dark. (B and C) Morning (B) and evening (C) anticipation indices were derived from the sleep profiles in (A). Wakefulness was calculated from 15-min activity bins using a custom R pipeline adapted from Vanessa-DAM outputs. Anticipation was quantified as the fraction of wakefulness in the 3 h preceding lights-on (morning anticipation) or lights-off (evening anticipation) compared to the total activity in that same period (day or night). Points represent individual flies with overlaid violins and boxplots. *p* values were computed by two-tailed *t* tests with Benjamini-Hochberg adjustment for multiple comparisons. Significance is denoted by a compact letter display (BH *p* < 0.05); genotypes that do not share a letter are significantly different from one another. (D) Estimated climbing velocity of core *Appl* alleles from a negative geotaxis assay. Climbing height was measured after 4 s and analyzed with a custom linear mixed model (LMM) R script. Individual velocity was calculated using measured height divided by 4 s; where nonresponders (velocity <0.293 cm/s) were excluded. This model included genotype as a fixed effect and cohort/date as random factors. Displayed are estimated marginal means ±95% CIs. Significance is denoted by a compact letter display (Tukey-adjusted *p* < 0.05); genotypes that do not share a letter are significantly different from one another. (A–C) n’s: *Appl*^d^ (36), *Appl*-T2A-GAL4 (14), CantonS (31), *w*^1118^ (15), *Appl*-RNAi (16), and RNAi Control (25); (D) n’s: *Appl*-T2A-GAL4 (99), CantonS (169), *Appl*^d^ (115), *w*^1118^ (112), *Appl*-RNAi (24), and RNAi Control (18).

To evaluate whether *Appl* loss also affects sustained motor output, we employed the negative geotaxis assay and analyzed climbing velocity using a linear mixed model. Nonresponders (velocity <0.293 cm/s) were excluded. Genotype was modeled as a fixed effect, and both cohort and date were modeled as random factors. Estimated marginal means indicated that *Appl*^d^ and *Appl*-T2A-GAL4 flies climbed significantly more slowly than their respective controls. To confirm that this locomotor deficit was strictly dependent on the loss of Appl rather than genetic background or CRISPR-insertion artifacts, we performed a pan-neuronal Appl knockdown. *Appl*-RNAi flies exhibited a significant reduction in climbing velocity that phenocopies the deficits seen in both *Appl*^d^ and *Appl*-T2A-GAL4 lines (Figure 7D).

These data collectively demonstrate that the loss of *Appl* impairs circadian anticipation, sleep stability, and locomotion. By validating these phenotypes across three distinct genetic models, this study highlights that *Appl*-T2A-GAL4 serves as a genetically precise null model, useful for future research on Appl functions. Since APP and Appl affect synaptic integrity and metabolic homeostasis, these disruptions in sleep and movement reinforce the conserved relationship between APP function, circadian regulation, and motor activity.

Building on these behavioral disruptions, we next asked whether the molecular pathways uncovered by our proteomic analysis represented isolated fly-specific effects or reflected a broader, conserved response to APP family dysregulation. Integrating our Appl-dependent proteomic signature with mouse and human AD datasets revealed a striking convergence on glycosylation, mitochondrial metabolism, and oxidative processes.^34^^,^^37^^,^^47^^,^^48^ Notably, the core factors Alg9, CG9240 (IMMP1L), and CG4594 (ECI1), which are significantly increased upon *Appl* loss in flies, show changes in well-established AD mouse models and human AD datasets (Figure 8A). To understand how these conserved regulators relate to APP biology, we generated a human protein-interaction network that incorporated the orthologs of these Appl hits. This analysis revealed that glycosylation machinery, mitochondrial factors, and oxidative enzymes all interact with APP through intermediary nodes (Figure 8B). Together, these findings position the *Appl* loss-of-function signature not as a *Drosophila*-specific phenomenon but as a conserved molecular program shared across flies, mice, and humans. This cross-species conservation provides a mechanistic foundation for understanding how APP family proteins regulate metabolic homeostasis across evolutionary distance.

**Figure 8:**
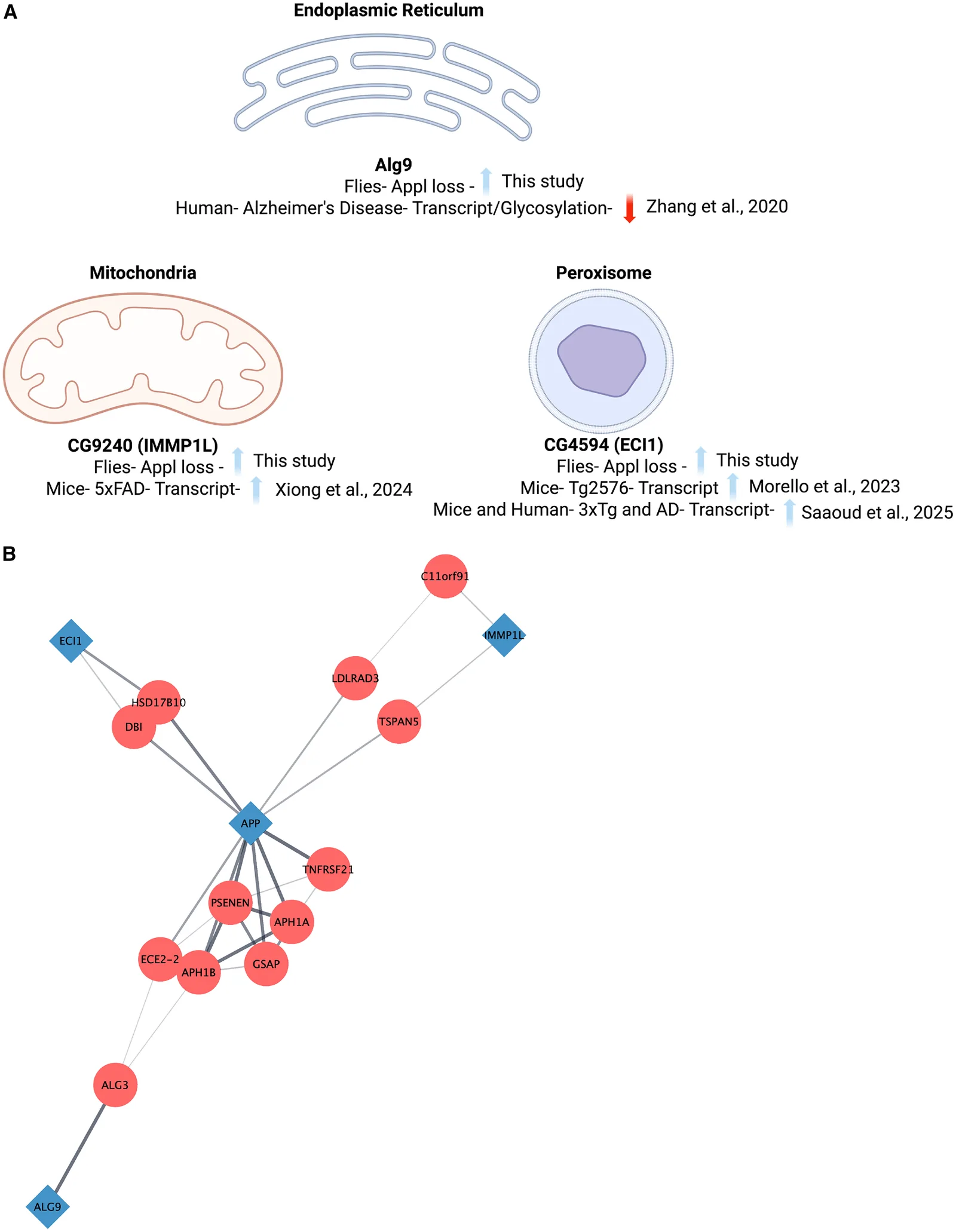
Cross-species convergence of Appl-regulated genes and pathway connectivity (A) Organelle-based summary schematic of conserved Appl-regulated genes. Evidence was compiled from this study (*Drosophila Appl* loss), mouse AD models (3×Tg, 5×FAD, and Tg2576), and human AD datasets. Target proteins are placed according to primary subcellular localization or functional compartment. For each gene, the *Drosophila* gene name is shown first followed by the human ortholog in parentheses, if different. Entries are formatted beginning with species-followed by model-followed by expression method-followed by direction of change and study citation. (B) Protein-protein interaction network linking Appl-regulated metabolic hits to the human APP interactome. Network generated using Cytoscape (v.3.10.3) using the STRING app with the following parameters—input: Alg9, IMMP1L, ECI1, APP; species: *Homo sapiens*; network type: full STRING network; confidence cutoff: 0.30; maximum additional interactors: 25. Layout was determined using Edge-weighted spring-embedded model using STRING combined score as the weight parameter. Edge width is continuously mapped to STRING score (thicker = higher confidence). Nodes corresponding to proteins homologs detected in this study, plus APP, are highlighted as blue diamonds. Linking proteins returned by STRING are shown as red circles. This network illustrates that these proteins reside in distinct organelles and are connected through various intermediary nodes.

## Discussion

This study provides an integrative molecular characterization of the widely used *Drosophila Appl*^d^ mutant, combining highly contiguous long-read genome assembly with quantitative proteomics to identify Appl-specific pathways separate from background effects. Our PacBio Revio assembly resolved two large deletions splitting the Appl locus across separate contigs and revealed additional X-linked variants likely caused by the high-dose irradiation used to generate *Appl*^d^ decades ago.^8^^,^^14^ Despite our limitations in refining contig localization, our data yield structural insights that clarify previous challenges in PCR-based mapping and confirm that *Appl*^d^ is not a straightforward deletion allele but a complex genomic rearrangement. While further physical mapping, such as fluorescence *in situ* hybridization (FISH) of polytene chromosomes or Hi-C mapping, may resolve the final coordinates of these translocated fragments, the identification of multiple background mutations in *Appl*^d^ suggests that such efforts would not mitigate the functional confounding effects inherent to this line. Consequently, we focused our efforts on establishing a clean genetic foundation through precise CRISPR engineering. By creating a set of CRISPR-engineered *Appl* mutants in clean genetic backgrounds, including a precise null-driver (*Appl*-T2A-GAL4) and an endogenously GFP-tagged allele, we produced genetically defined *Appl* loss-of-function models free of irradiation-induced confounders. Proteomic comparisons between these mutants and *Appl*^d^ identified a redefined Appl-dependent signature focused on glycosylation, protein trafficking, and mitochondrial metabolism, processes repeatedly implicated in APP biology and AD.^1^^,^^35^^,^^36^ By separating precise *Appl* loss-of-function effects from irradiation-induced background lesions, we both redefine the genetic basis of a key APP family member and establish translational framework that links *Appl* loss to molecular pathways disrupted in AD and APP mouse models.

### Conserved metabolic functions of Appl

The refined Appl-specific proteomic signature that emerged from our dual platform analysis highlights molecular pathways central to protein quality control and metabolic homeostasis. Among the most consistent hits, Alg9 encodes an α-1,2-mannosyltransferase essential for the N-linked glycosylation pathway, a post-translational modification critical for the folding, trafficking, and stability of both membrane and secreted proteins.^30^ Glycosylation defects have repeatedly been linked to AD pathogenesis, where altered glycan structures can influence APP processing, Aβ aggregation, and clearance.^35^^,^^36^ The second major candidate, CG4594, encodes a predicted mitochondrial isomerase with potential roles in fatty acid β-oxidation, a metabolic pathway increasingly linked to Aβ related toxicity and neurodegenerative disease.^33^ These changes align with the proposed roles of APP family members in regulating intracellular transport and turnover of synaptic proteins.^1^^,^^49^^,^^50^^,^^51^ Recent studies increasingly place APP-family proteins at the intersection of mitochondrial homeostasis, organelle trafficking, and metabolic regulation, supporting the idea that altered APP function can affect physiology independently of amyloid production.^52^^,^^53^^,^^54^^,^^55^ In this context, the identified mitochondrial protein CG9240, glycosylation protein Alg9, and oxidative protein CG4594 likely represent conserved APP-family functions rather than a fly-specific consequence of *Appl* loss.

### Clarifying the *Appl*^d^ allele

In parallel with defining the *Appl* loss-of-function signature, our analysis uncovered a distinct set of proteomic changes unique to the legacy *Appl*^d^ line, including a downregulation of three X-linked proteins (CG3156, Pdzd8, and Cyp311a1), which were each supported by both Orbitrap and timsTOF datasets. Genomic mapping revealed that *CG3156* harbors a frameshift mutation predicted to truncate the protein, while *Pdzd8* harbors a Val21Glu missense mutation near the N-terminus, potentially disrupting protein stability (Figure 6C; Figure S5). Mutations within transmembrane domains are prone to destabilizing protein trafficking or localization, and even a single substitution can interfere with stability or membrane insertion.^38^ These results suggest direct disruption caused by the irradiation-induced mutagenesis used to create *Appl*^d^.^8^ Given *Appl*^d^’s long-standing role as the *de facto* Appl null background in *Drosophila*, these mutations suggest that phenotypes attributed solely to *Appl* loss might partly result from secondary genetic lesions. Although our combined genomic-proteomic approach allows detection of such candidates, it has inherent limitations. Proteomic changes can occur indirectly via compensatory pathways rather than direct gene disruption, and our analysis cannot definitively assign gain-of-function or network effects to specific loci. Additionally, structural complexities at the *Appl* locus remain unresolved, and low-abundance or cell-specific proteomic changes may be overlooked in whole-head analysis. Nonetheless, by pinpointing specific background variants and providing genetically clean CRISPR null lines, we offer an immediate way for the field to re-examine *Appl*^d^-based findings under controlled genetic conditions. This dual approach of deconvolving legacy alleles and creating precise mutants can serve as a model for re-evaluating other historical genetic tools in *Drosophila* and beyond. For APP biology and AD research, this distinction is critical. Many conclusions drawn from *Appl*^d^ regarding APP family function, including synaptic maintenance, axonal transport, and behavioral phenotypes, may represent combined effects of *Appl* loss plus disruptions in background-specific pathways. Importantly, these findings do not refute the biological validity of previous research conducted in the *Appl*^d^ background. Many such studies have used transgenic rescues and independent RNA interference (RNAi) lines to rigorously confirm that the specific phenotypes are indeed *Appl*-dependent.^13^^,^^26^^,^^45^^,^^56^^,^^57^ Instead, our work provides a nucleotide-level and proteome-wide roadmap to further refine these results by identifying specific background factors that may have previously acted as unrecognized confounders in large-scale datasets. Our CRISPR-derived *Appl* null and reporter lines provide a cleaner platform for testing hypotheses regarding conserved mechanisms without confounding background lesions.

By integrating long-read genome assembly, precision genome engineering, and comparative proteomics, this work provides a molecular redefinition of the *Appl*^d^ allele and a genetically defined *Appl* loss-of-function proteomic signature. Beyond improving our understanding of APP family biology, this approach directly addresses the long-standing challenge in experimental genetics by revealing the hidden complexity of a legacy allele. In the context of developmental neurobiology, our findings link Appl to evolutionarily conserved pathways involved in glycosylation, protein trafficking, and mitochondrial metabolism—processes crucial for neuronal circuit maturation and connected to mechanisms implicated in human APP function and AD.^1^^,^^35^^,^^36^ Because these pathways are often disrupted in aging and neurodegenerative diseases, our results also establish a mechanistic link between early-life APP family functions and later disease processes, providing a unified framework for understanding APP biology throughout the lifespan. The CRISPR-generated *Appl* mutants not only enable detailed studies of these processes in *Drosophila* but also serve as a flexible genetic platform for cell-type-specific modulation, interaction analysis, and behavioral research. More broadly, our integrated genomic-proteomic approach offers a scalable model for re-evaluating widely used model alleles across different species, ensuring future research is based on genetics. By clarifying the genetic ambiguity of *Appl*^d^ and providing tools to move beyond its previous limitations, we open the door to advanced mechanistic studies of APP biology. This method enables exploration of conserved molecular pathways with greater accuracy and confidence.

### Loss of Appl disrupts activity and motor function

Our sleep and geotaxis experiments add a crucial behavioral dimension to the genomic and proteomic analyses, revealing how *Appl* loss manifests at the level of circadian outputs and motor performance. Under 12:12-h LD conditions, *Appl*-T2A-GAL4 and *Appl*^d^ show altered evening anticipation and fragmented sleep compared to controls (Figures 7B and 7C). These phenotypes are not unexpected given the pathways flagged by our proteomics. Proper glycosylation and mitochondrial trafficking are key to synaptic homeostasis, and their disruption is known to perturb clock outputs and sleep architecture.^58^^,^^59^ Loss of Appl likely compromises synapse function through deficits in N-linked glycosylation (Alg9) and fatty acid oxidation (CG4594), as indicated by our *Appl* loss-of-function signature (Figure 5D). Additionally, glial APP/Appl in *Drosophila* has been shown to regulate sleep-wake behavior and glutamate recycling, and Appl inhibition increases total sleep and prolongs bout duration.^60^ At the same time, *Appl*^d^ carries additional X-linked lesions, such as Pdzd8, that further impair metabolism and ER-mitochondria contacts, explaining why the phenotypes differ from *Appl*-T2A-GAL4 (Figures 6 and 7; Figure S8).

Our geotaxis data reinforce this view. Linear mixed effects modeling of climbing speed revealed significant slowing in both *Appl*-T2A-GAL4 and *Appl*^d^ when compared to controls, and non-responder analysis showed higher fractions of flies reaching the minimum cutoff (Figure S9). These motor deficits fit with the known role of Appl and APP in axon growth and synaptic maintenance. Overexpression of Appl increases synaptic bouton number at neuromuscular junctions, whereas null mutants have reduced bouton number and disrupted axonal transport.^11^^,^^61^^,^^62^ More importantly, the directionality of our effect mirrors recent work showing that reduced Pdzd8-mediated mitochondria-ER contacts in neurons slows age-related locomotor decline and improves fitness in *Drosophila*.^63^ While the concordant locomotor deficits across our CRISPR knockout, the legacy mutant, and RNAi knockdown models strongly validate the *Appl* loss-of-function phenotype, we cannot entirely rule out the possibility that inherent toxicity from the T2A-GAL4 expression system may also contribute to the severity of the behavioral deficits.^64^^,^^65^

Critically, the convergence of sleep and geotaxis phenotypes across our CRISPR-generated null and the legacy *Appl*^d^ line strengthens the assertion that *Appl* loss impairs circadian anticipation and motor ability. Divergences, such as opposite directions of evening anticipation index (EAI) between *Appl*-T2A-GAL4 and *Appl*^d^, make biological sense when considering additional X-linked mutations in *Appl*^d^. Given the structural rearrangements and background-linked proteomic disruptions we identified in *Appl*^d^, some phenotypes traditionally attributed to *Appl* loss in this line may require careful re-evaluation because of these confounding variants. Rather than diminishing the value of our data, this split, multi-model approach demonstrates how proteomics-informed genomics can separate gene-specific effects from background mutations. Overall, the behavioral assays and identified phenotypes provide a strong measure of Appl-dependent proteostasis and metabolism and align with established sleep and APP biology.^44^^,^^66^

### Summary

Taken together, our genomic, proteomic, and behavioral analyses define Appl as a central regulator of neuronal proteostasis whose disruption affects multiple organelles, alters organismal physiology, and whose downstream pathways are conserved across evolutionary distance. By uncovering the structural complexity and background mutations embedded within the historical *Appl*^d^ allele, and by establishing a suite of CRISPR mutants, we show that decades of work relied on a powerful but genetically complex model. Importantly, we also demonstrate that the core metabolic and behavioral phenotypes attributed to *Appl* loss were real, conserved, and mechanistically coherent. The convergence of glycosylation defects and mitochondrial and peroxisomal metabolism across flies, mouse AD models, and human AD datasets points to a shared metabolic axis sensitive to APP family disruption.^34^^,^^37^^,^^47^^,^^48^ These same pathways manifest as disrupted sleep architecture, impaired circadian anticipation, and reduced motor performance in Appl mutants, linking molecular dysfunction to organism-level outcomes. By integrating long-read genomics with cross-species proteomics and precise genetics, this work establishes a unified framework for how APP family proteins regulate neuronal function via organellar communication and metabolic balance. Rather than just revisiting a legacy allele, this study clarifies the genetic basis of a foundational model and provides insight into the molecular pathways that govern conserved APP family function.

### Limitations of the study

Several limitations should be considered when interpreting the findings of this work. First, our proteomic screen shows correlation where CG9240, Alg9, and CG4594 are reproducibly altered following *Appl* loss, but we have not tested whether manipulating them is necessary or sufficient to produce the sleep or locomotor phenotypes reported. Therefore, they are best regarded as candidate phenotypes rather than established readouts without resolved underlying circuitry. Proteomic and genomic measurements were made from whole heads, so cell-specific or low-abundance changes may have been diluted or missed. Our lines are also not fully isogenic, and although multi-genotype filtering strategies help to remove background-dependent differences, residual strain effects cannot be excluded, including the potential contribution of the T2A-GAL4 system itself.^64^^,^^65^ The structure of the *Appl*^*d*^ chromosome remains incompletely resolved, as no reads span the contig junctions. Definitive placement will require further mapping techniques. Furthermore, the convergence we observe with mouse and human AD datasets rests on cross-platform comparisons of transcript and protein measurements in different model systems where shared directionality of change does not establish a shared underlying mechanism.^34^^,^^37^^,^^47^^,^^48^

## Resource availability

### Lead contact

Requests for further information and resources should be directed to and will be fulfilled by the lead contact, Fernando J. Vonhoff (fvonhoff@umbc.edu).

### Materials availability

Fly stocks generated in this study will be deposited at the Bloomington Drosophila Stock Center upon publication.

### Data and code availability

• Raw long-read sequencing data have been deposited in the Sequence Read Archive (SRA) under accession SRR34799174 under BioProject PRJNA1299466. The *de novo* genome assembly has been deposited in GenBank under the accession JBTTGZ000000000. Raw mass spectrometry data have been deposited in PRIDE under accession PXD073417. Processed label-free quantification data for the Orbitrap and timsTOF experiments, supplemental datasets, and original western blot images are publicly available through Zenodo at https://doi.org/10.5281/zenodo.17751181.
• *Original code and analysis scripts generated for this study are publicly available through Zenodo at* https://doi.org/10.5281/zenodo.17751181 and on GitHub at https://github.com/zacharymsmith/Appl-d-_paper.
• Any additional information required to reanalyze the data reported in this paper is available from the lead contact upon request.

## Acknowledgments

Research reported in this publication was supported by the National Institute Of General Medical Sciences of the National Institutes of Health under Award Number R35GM163606. The content is solely the responsibility of the authors and does not necessarily represent the official views of the National Institutes of Health. We thank the Bloomington Drosophila Stock Center and the Drosophila Genomics Resource Center, supported by 10.13039/100000002NIH grant 2P40OD010949, for providing fly stocks and reagents used in this study. We are grateful to Philip Copenhaver for generously providing Appl antibodies, sharing technical insight, and providing feedback on the manuscript. We thank Bart De Strooper for providing molecular reagents. We also thank Claire Devine for generating CRISPR materials. We are especially grateful to Claudia Gualtieri for providing imaging experimentation during revision. We extend major thanks to Conor Jenkins for performing the mass spectrometry experiments that enabled the proteomic analyses central to this work.

## Author contributions

Z.M.S., conceptualization, methodology, formal analysis, data curation, investigation, writing, and reviewing and editing; F.J.V., conceptualization, funding acquisition, formal analysis, investigation, writing, resources, supervision, and reviewing and editing.

## Declaration of Interests

The authors declare no conflict of interests.

## Declaration of generative AI and AI-assisted technologies in the manuscript preparation process

During the preparation of this work, the author(s) (ZMS) used ChatGPT to polish the writing of the manuscript. After using this tool or service, the author(s) reviewed and edited the content as needed and take(s) full responsibility for the content of the publication.

## STAR★Methods

### Key resources table

**Table:** 

REAGENT or RESOURCE	SOURCE	IDENTIFIER
**Antibodies**		

Chicken anti-Appl (vs. YENPTYKYFEVKE cytoplasmic domain of Manduca Appl; cross-reactive to Drosophila Appl); pre-adsorbed vs. Appl[d] pupae. WB 1:1,000	P. Copenhaver (OHSU); gift	N/A
Rabbit anti-APP-c20 (clone B63.3; C-terminal APP; validation reagent only)	De Strooper Lab; gift	N/A
Mouse anti-ATP5A [15H4C4], mitochondrial marker. IF 1:100	Abcam	Cat# ab14748; RRID:AB_301447
HRP-conjugated AffiniPure Rabbit anti-chicken (secondary). WB 1:10,000	Proteintech	Cat# SA00001-6; RRID: AB_2890885
Goat anti-mouse IgG1 (γ1) Cross-Adsorbed, Alexa Fluor 568 (secondary). IF	Thermo Fisher (Invitrogen)	Cat# A-21124; RRID:AB_2535766

**Chemicals, Peptides, and Recombinant Proteins**		

RIPA lysis and extraction buffer	Thermo Fisher	Cat# 89901 (250 mL); also 89900 (100 mL)
Protease inhibitor (Pierce Mini Tablets, EDTA-free)	Thermo Fisher (Pierce)	Cat# A32953
Trypsin/Lys-C protease mix, MS grade	Promega	Cat# V5073
EveryBlot Blocking Buffer	Bio-Rad	Cat# 12010020
7.5% Mini-PROTEAN TGX Stain-Free gel	Bio-Rad	Cat# 4568024
Immun-Blot PVDF membrane, 0.2 μm	Bio-Rad	Cat# 1620184
SuperSignal West Atto Ultimate Sensitivity Substrate	Thermo Fisher	Cat# A38555
4× Laemmli sample buffer	Bio-Rad	Cat# 1610747
10× Tris/Glycine/SDS running buffer (SDS-PAGE)	Bio-Rad	Cat# 1610772
10× Tris/Glycine transfer buffer (Western transfer)	Bio-Rad	Cat# 1610771
Spectra Multicolor Broad Range protein ladder (MW markers)	Thermo Fisher	Cat# 26634
Methanol (PVDF activation)	Avantor/VWR	Cat# 97065–064
Paraformaldehyde (4% in PBS)	Standard	N/A
DAPI nuclear stain. IF 1:1,000	Sigma-Aldrich	Cat# D9542
Ethanol (DNA wash/head isolation slush)	VWR	Cat# EM-4450 S
Formic acid, MS grade (0.1%); glycerol; 1× PBS	Standard	N/A

**Critical Commercial Assays/Kits**		

Puregene Cell Kit, 8 × 10ˆ8 (HMW gDNA)	Qiagen	Cat# 158043
Pierce BCA Protein Assay Kit	Thermo Fisher	Cat# 23227
S-Trap micro spin column MS kit	ProtiFi (via Fisher)	Cat# NC1828286
No. 25 sieve, 710 μm (head isolation)	Cole-Parmer	Cat# EW-59987–12

**Deposited Data**		

*Appl*^d^ raw long-read HiFi genome data	This paper	SRA: SRR34799174; BioProject: PRJNA1299466
*De novo* genome assembly	This paper	GenBank: JBTTGZ000000000
Raw mass spectrometry data (Orbitrap + timsTOF)	This paper	PRIDE: PXD073417
Processed LFQ data, supplemental datasets, code	This paper	Zenodo: https://doi.org/10.5281/zenodo.17751181
Original code and analysis scripts	This paper	GitHub: https://github.com/zacharymsmith/Appl-d-_paper.git
Original western blot images	This paper	Zenodo: https://doi.org/10.5281/zenodo.17751181
*Drosophila melanogaster* reference genome dm6 (r6.62)	FlyBase	RRID:SCR_006549
UniProt reference proteome	UniProt	UP000000803/ID 7227; RRID:SCR_002380
Prior proteomic dataset (10-day flies)	Nithianandam et al.^26^	Nithianandam et al.^26^

**Experimental Models: Organisms/Strains (Drosophila melanogaster)**		

*Appl*^*d*^	BDSC	RRID:BDSC_43632
*w*^*1118*^	BDSC	RRID:BDSC_3605
Canton-S	BDSC	RRID:BDSC_64349
UAS-*Appl*-RNAi	BDSC	RRID:BDSC_28043
UAS-CD4-tdTomato	BDSC	RRID:BDSC_35841
*elav*-GAL4 (pan-neuronal driver; Figure 7D)	BDSC	RRID:BDSC_25750
PBac transposase line (cassette excision)	BDSC/source	RRID:BDSC_8285
*Appl*-T2A-GAL4 (null/GAL4 knock-in)	This paper (WellGenetics)	N/A
*Appl*-GFP (endogenous C-terminal sfGFP)	This paper (BestGene)	N/A

**Recombinant DNA**		

pU6-3-gRNA (sgRNA expression vector)	DGRC	DGRC #1362; RRID:DGRC_1362
pHD-sfGFP-ScarlessDsRed (donor plasmid)	DGRC/Addgene	DGRC #1365; Addgene #80811; RRID:Addgene_80811

**Software and Algorithms**		

Hifiasm v0.24.0	Cheng et al.^21^	RRID:SCR_021069
QUAST v5.0.2	Gurevich et al.^67^	RRID:SCR_001228
Racon v1.5.0	Vaser et al.^68^	RRID:SCR_017642
Minimap2 v2.28	Li, 2018^19^	RRID:SCR_018550
Merfin	Formenti et al.^69^	https://github.com/arangrhie/merfin
Jellyfish v2.3.0	Marçais and Kingsford^70^	RRID:SCR_005491
GenomeScope 2.0	Ranallo-Benavidez et al.^16^	RRID:SCR_017014
bcftools v1.21	Danecek et al.^71^	RRID:SCR_005227
SAMtools/HTSlib v1.21	Danecek et al.^71^	RRID:SCR_002105
Merqury v1.3	Rhie et al.^72^	RRID:SCR_022964
Longshot v1.0.0	Edge and Bansal^24^	RRID:SCR_025318
FreeBayes v1.3.6	Garrison and Marth^25^	RRID:SCR_010761
D-GENIES	Cabanettes and Klopp^23^	RRID:SCR_018967
BLAST+ (blastn) v2.16.0	Camacho et al.^73^	RRID:SCR_004870
Liftoff v1.6.3	Shumate and Salzberg^28^	RRID:SCR_026535
Proteome Discoverer v2.5	Thermo Fisher	RRID:SCR_014477
Sequest HT	Thermo Fisher	RRID:SCR_014594
Percolator	Käll et al.^74^	RRID:SCR_005040
Spectronaut v19.9	Biognosys	Biognosys; biognosys.com/software/spectronaut
Metascape v3.5	Zhou et al.^49^	RRID:SCR_016620
Cytoscape v3.10.3	Shannon et al.^75^	RRID:SCR_003032
VANESSA/Vanessa DAM	Ghosh and Sheeba^76^	https://github.com/orijitghosh/VANESSA-DAM
GraphPad Prism v10.4.2	GraphPad	RRID:SCR_002798
R/RStudio v4.3.2	R Core Team	RRID:SCR_000432/SCR_001905
R packages: ggplot2, lme4, lmerTest, emmeans, performance, tidyverse, ggpubr, RColorBrewer, scales	CRAN	See STAR Methods for versions

### Experimental model and study participant details

All experiments used *Drosophila melanogaster*. No human participants, vertebrate animals, or cell lines were used, and no ethical approval was required. Stocks obtained from the Bloomington Drosophila Stock Center were *Appl*^d^ (RRID:BDSC_43632), w^1118^ (RRID:BDSC_3605), Canton-S (RRID:BDSC_64349), UAS-*Appl*-RNAi (RRID:BDSC_28043), and UAS-CD4-tdTomato (RRID:BDSC_35841). Two lines were generated for this study: *Appl*-T2A-GAL4, a protein-null GAL4 knock-in made in a Canton-S background, and *Appl*-GFP, an endogenously C-terminally sfGFP-tagged allele. Both were validated by Sanger sequencing and western blotting. Flies were reared at 25°C on a 12 h:12 h light–dark cycle on molasses- and cornmeal-based food, as described in the STAR Methods.

*Appl* is X-linked, so males carrying a mutant allele are hemizygous and females carry one or two copies; sex is specified for each experiment below. Adults used for proteomics and western blotting were 3–5 days old, with Orbitrap and western samples pooled from 25 male and 25 female heads per replicate and timsTOF samples from five male heads per replicate. Genome sequencing used male heads. Imaging used first- and third-instar larval nervous systems. Climbing assays were performed on adults aged 1–5 days. Because *Appl*^d^ was generated by high-dose irradiation and is not isogenic with *w*^*1118*^ or Canton-S, comparisons involving *Appl*^d^ were interpreted alongside the CRISPR-engineered alleles.

### Method details

#### Fly stocks and husbandry

Fly stock lines were maintained at 25°C in a 12/12 light-dark incubator using standard culturing bottles and a molasses and cornmeal-based food as previously described.^77^

Isolation of the *Appl*-T2A-GAL4 chromosome was essential for testing whether the Appl locus was interrupted. Since *Appl*-T2A-GAL4 homozygous females are both rare and sterile (hemizygous males appear to be fertile), crosses were performed between *Appl*-T2A-GAL4 males and females of both *Appl*^d^ and *w*^*1118*^, as well as between *Appl*^d^ males and virgin *w*^*1118*^ females. Female XX progeny were collected to test the ability of various X chromosomes to produce detectable Appl.

#### Scarless Appl-GFP generation

The endogenously tagged *Appl*-GFP line was generated using scarless gene-editing protocols. Briefly, a donor plasmid containing the sfGFP sequence and a 3xP3-DsRed selection marker, flanked by transposon terminal repeats, was integrated into the *Appl* C-terminus via CRISPR/Cas9. To remove the selection marker, *Appl*-GFP+dsRed transformants were crossed with a line expressing the PBac transposase. Progeny from this cross were screened for loss of dsRed fluorescence, indicating successful excision of the cassette. The resulting line, *Appl*-GFP, was further validated by sequencing.

#### DNA extraction, preparation, and sequencing (Supplemental Folder 1)

High molecular weight DNA was extracted from ∼400 male *Appl*^*d*^ heads combined in one extraction using the Puregene Cell Core Kit (8 × 10ˆ8) following the supplemental protocol “Purification of archive-quality DNA from up to 30 *Drosophila melanogaster* using the Gentra Puregene Cell Kit” and following the protocol for extraction from 20–30 *Drosophila melanogaster*. Briefly, heads were frozen and isolated using an ethanol-dry ice slush, then lysed with a micro-pestle in 600 μL of Cell Lysis Solution. Once lysed, the solution was incubated at 65°C for 15 min, incubated with RNase A Solution for 50 min at 37°C, cooled to room temperature briefly with ice water, and protein was precipitated using 200 μL of Protein Precipitation Solution and vortexed vigorously and centrifuged (15,000 g for 3 min) to form a protein pellet. The supernatant was removed and added to a fresh tube with 600 μL of isopropanol. The mixture was gently mixed by inversion 50 times, then centrifuged to pellet the DNA at 15,000 g for 1 min. The supernatant was discarded, and the tube was carefully dried of any remaining isopropanol. The DNA pellet was washed with 600 μL of ethanol, re-pelleted by centrifugation at 15,000 g for 1 min, supernatant was discarded, and the pellet was dried at room temperature for 15 min. The pellet was rehydrated with 100 μL of DNA Hydration Solution, incubated at 65°C for 1 h to dissolve the pellet, and then shaken at room temperature overnight. Concentration was determined using a Nanodrop spectrophotometer and subsequently sent to Azenta for further quality control, preparation (AMPure bead cleanup), and PacBio Revio sequencing. Analysis report available in Supplemental Files (Folder 7).

#### Genome assembly and quality assessment (Supplemental Folder 1–2)

Long-read (HiFi) sequencing data from the PacBio Revio platform was provided in a FASTQ file (*Appl*^*d*^*M*.hifireads.fastq.gz) for input into Hifiasm (v0.24.0) for assembly using the dm6 reference genome. Reads were uploaded to the Sequence Read Archive (SRA) under the BioProject Accession number: PRJNA1299466. The primary assembly was used for all downstream analyses. Assembly quality was initially evaluated using QUAST (v5.0.2) using dm6 as a ref.^67^

To improve base-level accuracy, two rounds of polishing were performed using Racon (v1.5.0).^68^ Reads were mapped to the assembly using Minimap2 (v2.28) and recommended gap scoring parameters.^19^ After each polishing round, the assembly quality was evaluated using QUAST, where improvement plateaued after the second round.^67^

K-mer informed polishing was performed next using Merfin.^69^ Jellyfish (v2.3.0) was used to count 21-mers from the raw HiFi reads to generate histograms of peak coverage information.^70^ The histogram was visualized using GenomeScope 2.0^16^. These k-mer statistics were used with Merfin to refine the Racon2 assembly using variants identified with bcftools (v1.21).^69^^,^^71^ The final polished assembly was generated using bcftools consensus to compile the variants into the final assembly.^71^ Post polishing, the assembly was reevaluated using QUAST and Merqury (v1.3).^67^^,^^72^

#### Variant calling and structure analysis (Supplemental Folder 3–4)

Variants were called using both Longshot (v1.0.0) which used standard settings because it is optimized for long read data, and FreeBayes (v1.3.6) with parameters adjusted for long read data (minimum coverage: 5, minimum alternate count: 2, minimum alternate fraction: 0.2, minimum base quality: 10, minimum mapping quality: 20).^20^^,^^24^^,^^25^ VCF files were compressed with bgzip and indexed with tabix (htslib (v1.21)).^78^ Statistics were generated using bcftools and visualized.^71^ Overlapping and unique variants per caller were identified using the bcftools isec (v1.21) command.^71^ Distribution and count of variants were obtained by parsing the resulting VCF sites file, which was normalized to the reference (dm r6.62) chromosome lengths. Variant counts and frequency were graphed using GraphPad Prism 10 (v10.4.2(534)). Reads were mapped to the reference, and coverage was compiled into 1 Mb coverage bins that were visualized using Samtools (v1.21) and plotted in RStudio (v4.3.2) with ggplot2 (v3.4.4).^79^

The overall contig structure relative to the reference genome (dm r6.62) was visualized using D-GENIES to show both the assembly contiguity and the contig order based on synteny. Plots were generated using standard settings. Contigs were arranged in the GUI to highlight the contig placement using the sort function.

BLASTn (v2.16.0+) was used to align selected genes to the final assembly and PseudoX. Local blast databases were generated using makeblastdb, and alignments were performed with default parameters.^73^

The structural arrangement of contigs ptg000024L and ptg000067L was analyzed by extracting each read from the final assembly, aligning it to each contig using Minimap2 and Samtools. These two contigs were assembled using synteny from the reference for the order and orientation of the contig assembly. PseudoX was constructed by extracting ptg000067L, adding a 20-nucleotide spacer, and adding the reverse complement of ptg000024L. Liftoff (v1.6.3) was used to transfer annotations from the reference genome (dm6 r6.62 GTF) (using include copies, exclude partial, and specifying gene/transcript features).^28^

#### Protein extraction

Flies aged 3–5 days were flash-frozen in liquid nitrogen and vortexed to remove their heads. Heads were removed from bodies and wings using No. 25 sieves (Cole-Parmer). The heads of 25 males and females were combined. The frozen heads were weighed, and RIPA (Thermo) supplemented with a protease inhibitor cocktail (Pierce) was added at a concentration of 7.5 μL per milligram of tissue. The tissue was then homogenized with mini pestles by hand. Homogenate was incubated at 4°C for 30 min, followed by 20 min of centrifugation (at 4°C) to clarify the lysate. The supernatant was collected and quantified using a Pierce BCA protein Assay Kit (Thermo). The supernatant was stored at −20°C until use.

#### Protein electrophoresis and western blotting (Supplemental Folder 5)

Protein extracts were mixed to an equal volume with Laemmli loading buffer and deionized water, boiled briefly at ∼100°C for 2 min, and 40 μg of each sample was loaded into a 7.5% TGX stain-free gel (BioRad). Each lane represents biological replicates of separately extracted groups of aged flies. Electrophoresis was run in running buffer (Bio-Rad) at 100 V for ∼1 h, until the loading front reached the bottom of the gel. Proteins were transferred using transfer buffer (BioRad) onto an Immun-Blot 0.2 μm PVDF membrane (BioRad) that was previously activated for 30 s in methanol at a constant voltage of 100 V for 1 h. Post-transfer, the membrane was briefly washed with deionized water and dried at room temperature for 1 h. Membrane was subsequently reactivated in methanol for 30s, blocked in EveryBlot blocking buffer (BioRad), and incubated overnight at 4°C in primary solution (1:1 k chicken anti-Appl pre-adsorbed against fixed *Appl*^d^ pupae (Philip Copenhaver) in EveryBlot blocking buffer (Figures 4A and 4B)). This chicken anti-Appl antibody was generated against the sequence YENPTYKYFEVKE within the cytoplasmic domain of *Manduca* Appl, with cross-reactivity to *Drosophila* Appl.^80^^,^^81^ A secondary antibody (1:10 k rabbit-*anti*-chicken (Proteintech) in EveryBlot blocking buffer (Figures 4A and 4B)) was subsequently added and incubated at room temperature for 1 h. As an independent validation of antibody specificity, additional blots were probed with rabbit anti-App-c20 (B63.3 antibody from the De Strooper Laboratory) which recognizes the C-terminal region of APP.^82^ Cross-reactivity of B63.3 with *Drosophila* Appl was confirmed by personal communication with B. De Strooper and further supported in-house by loss of immunoreactivity in protein null Appl lines (blots not shown). Membrane was imaged using a GelDoc XR (BioRad) and SuperSignal West Atto Ultimate Sensitivity Substrate. Images were processed and saved using Image Lab Software (Bio-Rad) (Supplemental Folder 5).

#### Thermo orbitrap eclipse proteomics sample preparation

Samples were prepared for MS analysis using the S-Trap micro-MS kit (Protifi, Fairport, NY, USA) as previously described.^83^ Flies aged 3–5 days were flash-frozen in liquid nitrogen and vortexed to separate heads, which were isolated using No. 25 sieves (Cole-Parmer, Vernon Hills, IL, USA). For each genotype, three independent replicates were prepared, with each replicate containing 25 male and 25 female heads (50 total). Lysates were generated in RIPA buffer (Thermo Fisher Scientific, Waltham, MA, USA) supplemented with protease inhibitor cocktail (Pierce) at 7.5 μL per mg of tissue. Homogenates were incubated at 4°C for 30 min, centrifuged for 20 min at 4°C, and clarified supernatant was collected. Protein concentration was determined using a BCA assay (Thermo Fisher Scientific). Aliquots of 100 μg protein per replicate were processed with the S-Trap kit and digested overnight with Trypsin Lys-C (Promega, Madison, WI, USA) at 10 μg per sample. Peptides were eluted using standard buffers and stored at −20°C until drying and reconstitution.

#### Thermo orbitrap eclipse proteomics mass spectrometry acquisition and data analysis

Proteomic acquisition was performed on a Thermo Orbitrap Eclipse with a FAIMS interface, coupled to an Ultimate3000 HPLC, following our previously published workflow.^83^ Briefly, 5 μL (1.5 μg total) were injected onto a PepMap 100 trap column (75 μm × 2 cm, Thermo Fisher) and separated on an Easy-Spray analytical column (75 μm × 75 cm, 2 μm C18, Thermo Fisher) using a 200 min gradient (5–90% B). The Orbitrap Eclipse was operated in positive polarity and data-dependent acquisition mode, with FAIMS CVs set to −50, −65, and −85 V. Full MS scans were acquired at 240,000 resolution (m/z 350–2000). Data-dependent MS2 was performed by HCD (28% CE, 1 m/z isolation) with dynamic exclusion of 60 s.

Raw data were analyzed in Proteome Discoverer v2.5 (Thermo) using Sequest HT against the *Drosophila melanogaster* UniProt reference proteome (UP000000803, Dec 2024).^84^^,^^85^^,^^86^ Parameters included MS1 tolerance 10 ppm, MS2 tolerance 0.6 Da, static carbamidomethyl (C), and variable Met oxidation and protein N-terminal acetylation. FDR control was applied using Percolator (0.01/0.05 high/medium), PTM localization with IMP-ptmRS, and label-free quantification with Minora.^87^^,^^88^^,^^89^

#### Bruker timsTOF sample preparation

Sample digestion followed the same S-Trap protocol as described above, with input adjusted to 16 μg protein from five male heads per replicate (three replicates per genotype). Digested peptides were dried in a cold trap vacuum centrifuge and resuspended to 200 ng/20 μL in 0.1% formic acid (MS grade). Peptides were loaded onto Evosep Tips and analyzed on an Evosep One system using the 30 samples/day method with an Evosep performance column (15 cm × 150 μm, 1.5 μm).

#### Bruker timsTOF proteomics mass spectrometry acquisition and data analysis

Mass spectrometry was performed on a Bruker timsTOF Ultra 2 under DIA-PASEF acquisition. Source settings: positive mode, 4500 V capillary, endplate offset 800 V, nebulizer 0.5 bar, dry gas 8.0 L/min, dry temperature 200°C. Scans were acquired across m/z 100–1700 with an ion mobility range 1/K0 0.7–1.3 and a ramp time of 120 ms.

Data were processed in Spectronaut v19.9 (Biognosys, Zurich, Switzerland) in library-free mode, searched against the *Drosophila melanogaster* UniProt reference proteome (ID: 7227).^86^^,^^90^

#### Proteomics visualization (Supplemental Folders 6–8)

Visualization of pathway protein-protein interactions (PPIs) was performed in Metascape (v3.5) using significantly differentially expressed proteins as input.^49^ A background gene list was provided as additional input. Cytoscape (v3.10.3) was used to visualize and annotate PPI pathways, where color was mapped to fold change (blue representing an increase and red representing a decrease). Clusters were labeled by a summary of GO terms for each pathway, individual terms, and values available in supplements (Supplemental Folder 6–8).

A comparison between our Orbitrap data on 3-5-day-old flies and previously published data on 10-day-old flies was performed by removing hits not found in either dataset or only found in one, and sorting by expression levels (Supplemental Folder 9). Significant protein lists from both datasets were compared and combined using VLOOKUP and then filtered to retain proteins found in both. These proteins were grouped into four categories based on their Log(2) fold change (FC) expression pattern.

#### CRISPR-Cas9 design and line generation

Various fly lines were designed using distinct strategies. For the *Appl*-GFP construct for sgRNA (pU6-3-gRNA; addgene stock #1362) and donor plasmid (pHD-sfGFP-ScarlessDsRed and addgene stock #1365) were processed in-house, prepped, and sent to BestGene Inc. for injection and selection of transformants. Validation for these lines was performed in-house with Sanger sequencing and western blotting. For the *Appl*-T2A-GAL4 line, design, injection into Canton-S background, and screening for transformants were all performed by WellGenetics, Taiwan. Lines have been validated both externally through sequencing and internally through western blot analysis to confirm disruption of Appl expression. Validation is represented in Figure 4 and Supplemental Folder 5.

#### Nervous system dissection, immunostaining, and confocal imaging

Third instar larval nervous systems were dissected in 1× PBS, fixed in 4% paraformaldehyde, and mounted in 100% glycerol unless otherwise specified, as previously described.^91^^,^^92^^,^^93^ Immunostaining of third-instar larval brains and ventral nerve cords was performed using established *Drosophila* larval brain staining protocols.^94^ Briefly, dissected tissue was fixed, washed, blocked, and incubated with anti-ATP5A antibody [15H4C4] mitochondrial marker (Abcam: ab14748; 1:100). After washing, samples were then incubated with anti-mouse Alexa Fluor 568 secondary antibody, washed three times, and counterstained with DAPI (1:1000) for 1h at room temperature. Samples were then washed again in PBS and mounted in 100% glycerol. These preparations were used to visualize *Appl*-GFP signal together with mitochondrial (ATP5A) and nuclear markers in third-instar larval nervous tissue.

First instar larval brains were dissected as previously described, fixed in 4% paraformaldehyde for 20 min, washed in PBS for 20 min, and mounted in 100% glycerol.^94^ First instar preparations were imaged for GFP signal without additional immunostaining.

Confocal imaging was performed using a Zeiss Axio Observed Z1/7 microscope with a Plan-apochromat 63×/1.40 oil objective. Third-instar larval preparations were imaged using 405 nm, 488 nm, and 561 nm excitation for DAPI, *Appl*-GFP, and ATP5A (AF568), respectively. First instar larval *Appl*-GFP samples were imaged using Airyscan. Scale bars on Figures S2B and S2C are 10 μm.

#### Sleep/activity recording and analysis

Adult flies were recorded in 12:12 h light-dark (LD) conditions at 25°C in Drosophila Activity Monitors (Trikinetics). Activity files were analyzed using Vanessa DAM and exported in 15 min sleep bins in a per-fly time series that was processed in RStudio (v4.3.2).^76^ For each fly we computed wakefulness (awake = 1-asleep), where asleep is the fraction of sleep per 15 min bin in the source file. Time was converted to hours and wrapped to a 0-24 h index. Morning anticipation index (MAI) was defined as the proportion of night wakefulness occurring in the last 3 h before lights on (21-24 h) divided by the total wakefulness during lights off (12-24 h) and evening anticipation index (EAI) was defined as the portion of day wakefulness occurring in the last 3 h (9-12 h) before lights off (0-12 h) similar to previous analysis.^95^

#### Negative geotaxis

Climbing was assayed with a standard negative geotaxis protocol. Briefly, flies aged 1–5 days from selected genotypes were placed in climbing vials 1 h before testing. Climbing was recorded across four replicates, separated by 1 min of recovery between trials. Climb height was recorded for each individual at 4s after cessation of tapping.

Primary inference used only responders, and we fit a linear mixed-effects model (LMM) with genotype as a fixed effect and date and nested cohort as random effects, similar to our previously published work.^96^ Model summaries and diagnostics are exported. Estimated marginal means (EMMs) and 95% CIs were obtained with emmeans (v1.11.2.8) and pairwise Tukey contrasts were computed with significant comparisons overlaid on the plots. Analyses were performed in RStudio with tidyverse (v2.0.0) and ggpubr (0.6.2) for sleep, and for climbing: lme4 (v1.1.37), lmerTest (v3.1.3), emmeans (v1.11.2.8), performance (v0.15.2), and plotting packages RColorBrewer (v1.1.3) and scales (v1.4.0) were used.^97^

### Quantification and statistical analysis

Statistical details for each experiment, including the test used, sample size, and definition of replicates, are noted in the corresponding figure legends and method details sections. Significance was defined as *p* < 0.05 throughout, with false discovery rate correction applied where multiple comparisons were performed.

Analyses were carried out using standard software. Proteomic false discovery rates were controlled in Proteome Discoverer v2.5 using Percolator, with post-translational modification localization by IMP-ptmRS and label-free quantification by Minora; timsTOF data were processed in Spectronaut v19.9. Variant counts and distributions were plotted in GraphPad Prism v10.4.2. Sleep and climbing data were analyzed in R/RStudio v4.3.2. Sleep comparisons used two-tailed t-tests with Benjamini-Hochberg correction, and flies dead or immobile for more than two days were excluded. Climbing was analyzed with a linear mixed-effects model (lme4, lmerTest) with genotype as a fixed effect and date and nested cohort as random effects; estimated marginal means and pairwise Tukey contrasts were obtained with emmeans. Non-responders were defined as flies below 0.2932 cm/s and reported with binomial 95% confidence intervals.
