# Supplementary material for: Drosophila Appl loss disrupts proteins in mitochondrial oxidative and glycosylation pathways conserved in Alzheimer's disease
Source: iScience. 2026 Oct 12;29(11):117781. doi: 10.1016/j.isci.2026.117781 (PMC13682386; doi:10.1016/j.isci.2026.117781)
Supplement: Supplementary file 1 [file mmc1.pdf]

iScience, Volume *29*

Supplemental information

Drosophila Appl loss disrupts proteins in mitochondrial oxidative and glycosylation pathways conserved in Alzheimer's disease

Zachary M. Smith and Fernando J. Vonhoff

Figure S1: Blast mapping reveals fragmentation of the *Appl* locus in the *Appl*^{d} assembly.

A

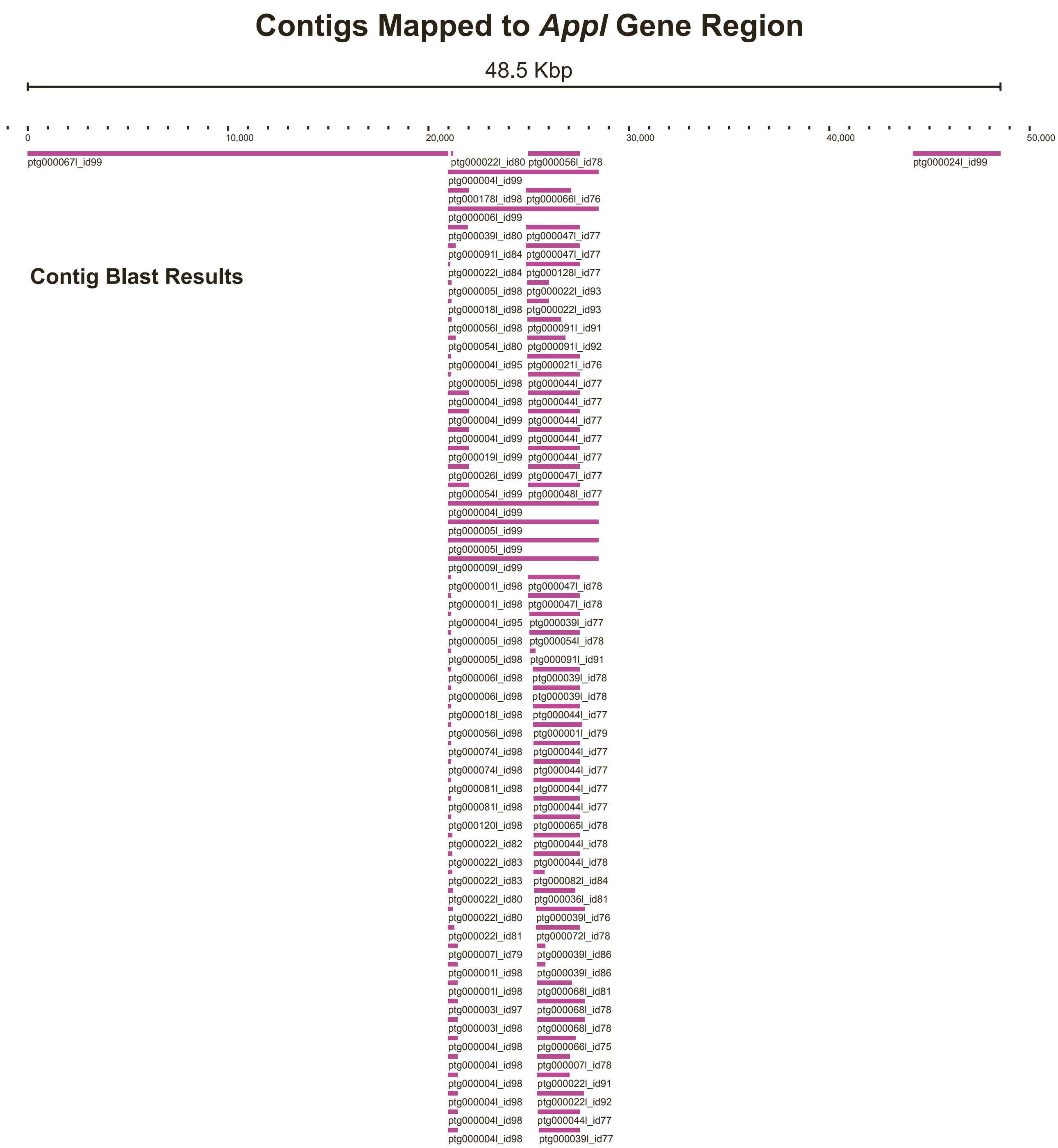

A- To assess the distribution of the Appl locus throughout the assembly, BLASTn was used to search the Appld assembly as a query against the Appl locus. Output from the search was converted to BED format using awk that extracted coordinates and renamed with percent identity (_id(%)). The resulting file was sorted, indexed, and visualized in Jbrowse2. The top track shows the reference Appl region obtained from Flybase, and the lower track represents contig hits from the assembly. Two main contigs contain a majority of the non-repetitive Appl coding sequence (ptg000067l and ptg000024l). Additional hits show the likely complexity and repetitiveness of other regions and also the lack of coverage. The figure shows some of the hits; more are hidden but available for visualization.

Figure S2: Validation of CRISPR-engineered *Appl* alleles.

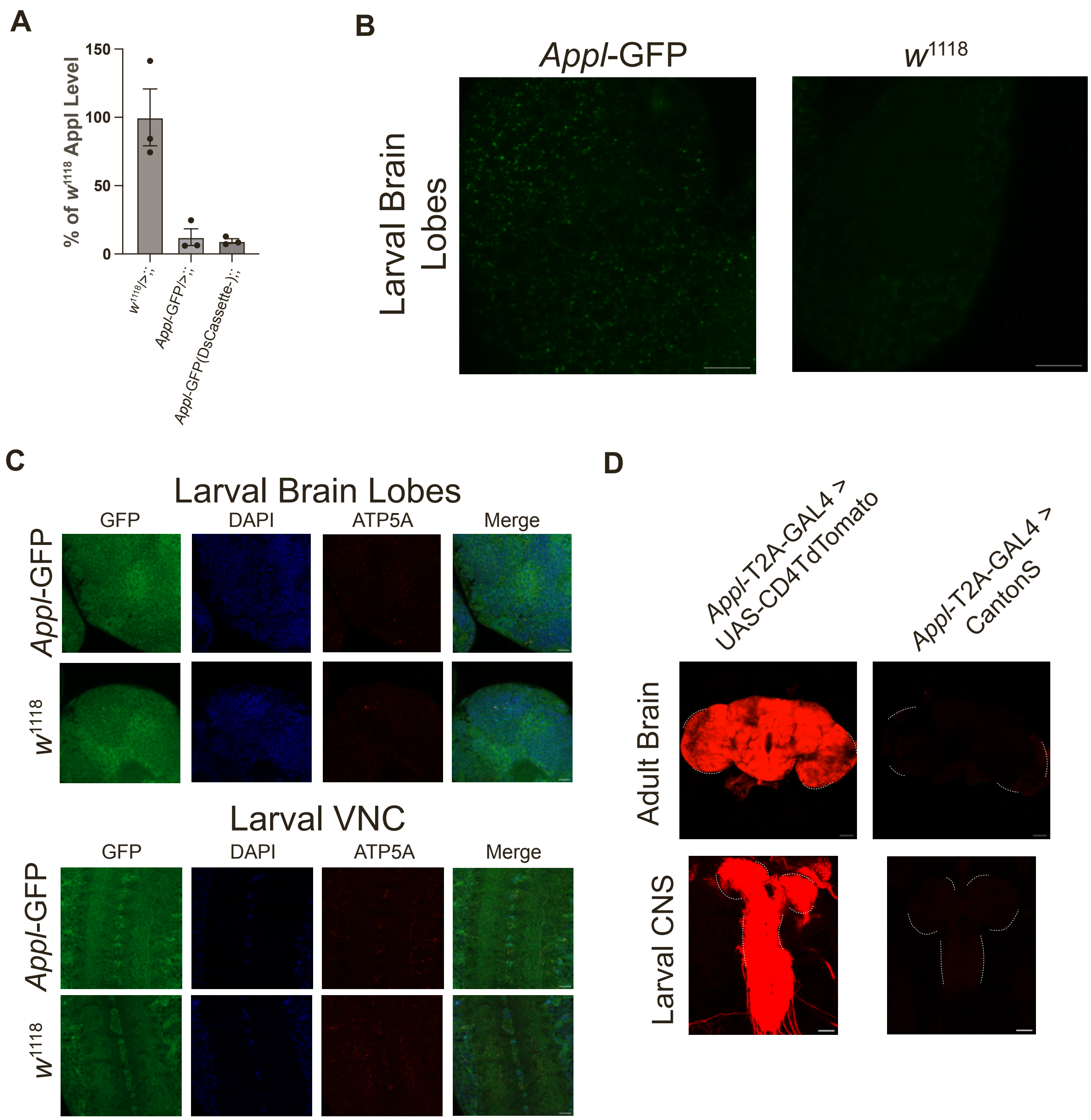

A- Quantification of western blots from Figure 4A of Appl-GFP mutants. Expression was quantified using Image Lab (Bio-Rad). Error bars represent the standard error of the mean (SEM). Expression of Appl is lower in the GFP-tagged lines with and without the DsRed cassette present. Scarless cassette removal is explained briefly in the methods. B- Confocal images of first-instar larval brain lobes showing GFP signal from Appl-GFP animals (left) compared to w1118 controls (right) imaged without antibody amplification. Images acquired and displayed with identical settings. Scale bars are 10µm. C- Confocal images of third-instar larval brain lobes (top) and ventral nerve cord (bottom) from Appl-GFP and w1118 control animals. Samples show endogenous (non-stained) Appl-GFP levels, ATP5A staining (red) for mitochondria and DAPI staining (blue) for nuclei as well as merged images (right). Scale bars are 10µm. D- Validation of Appl-T2A-GAL4 reporter activity. Appl-T2A-GAL4 crossed to Canton S shows minimal tdTomato background, whereas Appl-T2A-GAL4 crossed to UAS-CD4::tdTomato drives robust tdTomato expression in the adult brains (top) and third-instar larval nervous system (bottom). Scale bars are 50µm.

Figure S3: Orbitrap proteomics reveals broad proteomic dysregulation in *Appl^{d}* flies.

A

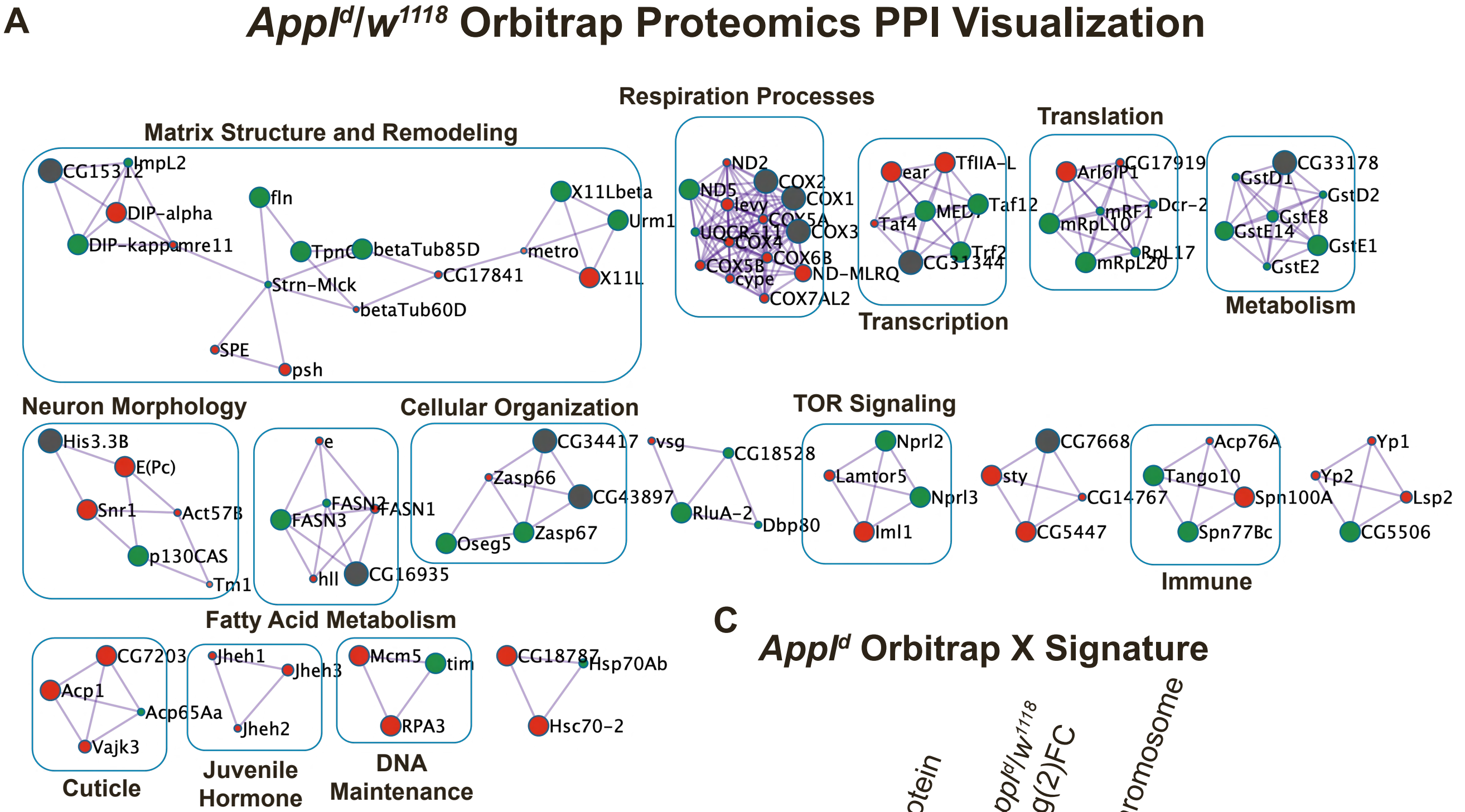

B

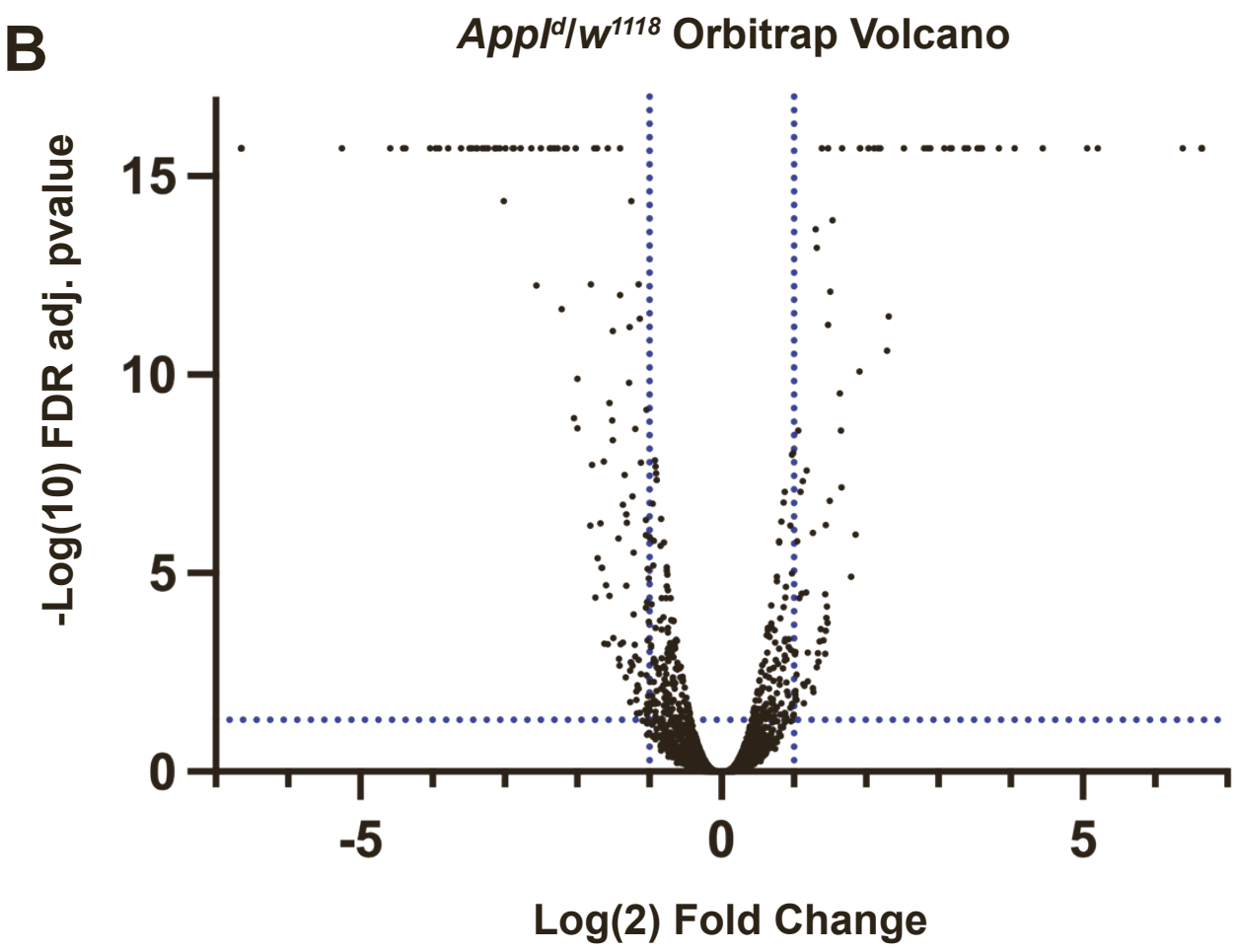

C

Appl^{d} Orbitrap X Signature

Protein	Appl^{d}/w^{1118} Log(2)FC	Chromosome
**CG17841**	-0.788	X
**COX6B**	-0.760	X
**DIP-alpha**	-6.644	X
**Lamtor5**	-1.215	X
**Nprl2**	6.644	X
**psh**	-1.231	X
**RPA3**	-6.644	X
**RpL17**	0.816	X
**Tango10**	6.644	X
**Trf2**	6.644	X
**X11L**	-3.604	X
**X11Lbeta**	6.644	X
**Yp1**	-0.564	X
**Yp2**	-0.622	X

A- Protein-protein physical interaction network generated from significantly ($P<0.05$) changed proteins in *Appl^{d}* as compared to w^{1118} created in Metascape. Clustering labels are a summary of various GO terms (see supplemental for full exact terms). Nodes are colored by fold change, upregulated in green (FC >= 1), downregulated in red (FC <=1), grey coloring denotes nodes not found in our data or missing expression value. Node size corresponds to the -Log10(adj. Pval) with larger size being more significant. B- Volcano plot of proteins detected comparing *Appl^{d}* vs w^{1118}. X axis indicates Log2(FC); Y-axis shows –Log10(adj. Pval). Blue vertical lines indicate $\log_2$FC ±1 and a horizontal blue line is at $P<0.05$ (-Log10(adj. Pval)=~1.3). C- Table of proteins found to be part of the physical interaction network and located on the X-chromosome. List highlights potential candidates for disruption during the creation of Appld chromosome. Visualization of pathway protein-protein interactions (PPIs) was performed in Metascape (v3.5) using significantly differentially expressed proteins as input[49]. A background gene list was provided as additional input. Cytoscape (v3.10.3) was used to visualize and annotate PPI pathways, where significance was mapped to node size (larger nodes indicating greater significance) and color was mapped to fold change (green representing an increase in *Appl^{d}* compared to w^{1118}, and red representing a decrease). Clusters were labeled by a summary of GO terms for each pathway, individual terms, and values available in supplements. A comparison between our data and previously published data on 10-day-old flies was performed by removing hits not found in either data set or only found in one, and sorting by expression levels. Significant protein lists from both datasets were compared and combined using VLOOKUP and then filtered to retain proteins found in both. These proteins were grouped into four categories based on their Log(2) fold change (FC) expression pattern.

Figure S4: Orbitrap proteomics identifies differentially expressed proteins carrying annotated post-translational modifications in *Appl^d* flies.

A

Appl^d/w^1118 Orbitrap Significant Phospho/Acetyl PTMs

Protein	Appl^d/w^1118 Log(2)FC	Appl^d/w^1118 -Log(10) FDR adj. pval	Modification
fln	6.644	15.708	Phospho [T49(99.5)]
RluA-2	6.644	15.708	Phospho [T359(96.7)]
unc-104	6.644	15.708	Phospho [T1406(85)]
pyd	6.644	15.708	Phospho [S351(100); T362(94.9)]
G6P	6.644	15.708	Acetyl [N-Term]
sls	1.916	15.708	Phospho [S6283(100); S15906(89.7)]
sls	1.438	4.464	Phospho [S6283(100); S12926(89.7)]
rdgB	1.198	2.279	Phospho [S375(100); S713(100)]
CG7029	0.900	1.360	Phospho [T846(82)]
Mal-B2	0.873	7.048	Phospho [S242(100)]
CG10550	0.646	3.423	Acetyl [N-Term]
bt	0.628	3.438	Phospho [S700(99.8)]
Gart	0.546	2.519	Phospho [S814(98.9)]
ninaE	0.546	2.012	Phospho [S357(88.6)]
shot	0.521	1.309	Phospho [S2342(97.8); S5326(82.2)]
ninaC	-0.445	1.504	Acetyl [N-Term]
up	-0.452	1.567	Phospho [T28(100); T332(100)]
Zasp66	-0.454	1.579	Phospho [T344(79.6)]
Map205	-0.468	1.545	Acetyl [N-Term]
CG8927	-0.472	1.603	Phospho [S78(97.7)]
Nep7	-0.512	2.115	Phospho [S38(99.9)]
CG17224	-0.512	1.372	Phospho [S145(100)]
Mf	-0.530	2.289	Phospho [S219(88); S297(100); S299(100)]
Pfrx	-0.540	2.392	Acetyl [N-Term]
CG8908	-0.582	1.556	Phospho [T1233(80.5)]
CG1674	-0.597	1.713	Phospho [S843(84.9)]
Yp2	-0.622	3.289	Phospho [S173(100)]
FASN1	-0.625	3.329	Phospho [S2183(99.3); S2186(81.6)]
up	-0.666	1.341	Phospho [T323(100)]
puml	-0.690	1.755	Phospho [S340(84.1)]
CG3950	-0.692	2.970	Phospho [S496(86.8); S502(99.7); S734(97.2)]
Zasp66	-0.743	2.799	Phospho [T181(79.6)]; Acetyl [N-Term]
bnb	-0.747	4.959	Phospho [S192(100)]
Cpr97Ea	-0.828	4.363	Phospho [Y144(100)]
Tm1	-0.851	2.599	Acetyl [N-Term]
Bacc	-0.925	7.847	Phospho [S55(98.4)]
CG16772	-0.988	2.262	Phospho [S251(100)]
hll	-1.009	1.590	Acetyl [N-Term]
M7BP	-1.165	2.168	Phospho [S1683(99.3)]
CG8154	-1.246	14.367	Phospho [S253(100)]
Gbp2	-1.283	9.796	Phospho [S169(91); S302(100)]
CG18304	-1.429	5.869	Phospho [S1592(100)]
Mf	-1.501	3.364	Phospho [S219(88); S245(100); S247(100)]
AMPdeam	-1.716	5.378	Phospho [S47(100)]
Ndae1	-3.307	15.708	Phospho [T102(100)]
pyd	-6.644	15.708	Phospho [S634(100); T645(94.9)]
anon-WO03002137.1	-6.644	15.708	Phospho [S376(100)]
CG13293	-6.644	15.708	Phospho [S289(94.8)]
ear	-6.644	15.708	Phospho [S155(98.2)]
CG6982	-6.644	15.708	Acetyl [N-Term]

A- Table summarizing proteins significantly altered in *Appl^d* relative to *w^1118* that also contained PTMs (phosphorylation or acetylation) as identified by Orbitrap proteomics. Values represent Log2(fold change) and –Log(10) false discovery rate adjusted p-value. Selected proteins of interest are notable, including rdgB (PI-transfer protein; phospho-S375/S713), Zasp66 (Z-disk/actin anchoring; phospho-T181), sls (titin-like protein; multiple phospho sites), fln (filamin; phospho-T49), pyd (junctional scaffold; phospho-S634/T645), and Map205 (microtubule bundling protein; N-terminal acetylation) which are all implicated in cytoskeletal dynamics. This dataset is limited to an *Appl^d* vs *w^1118* comparison. Enrichment for phototransduction pathways was also observed but may reflect the white-eyed background of *w^1118* rather than Appl loss.

Figure S5: Structural modeling predicts local effects of the Pdzd8 V21E variant.

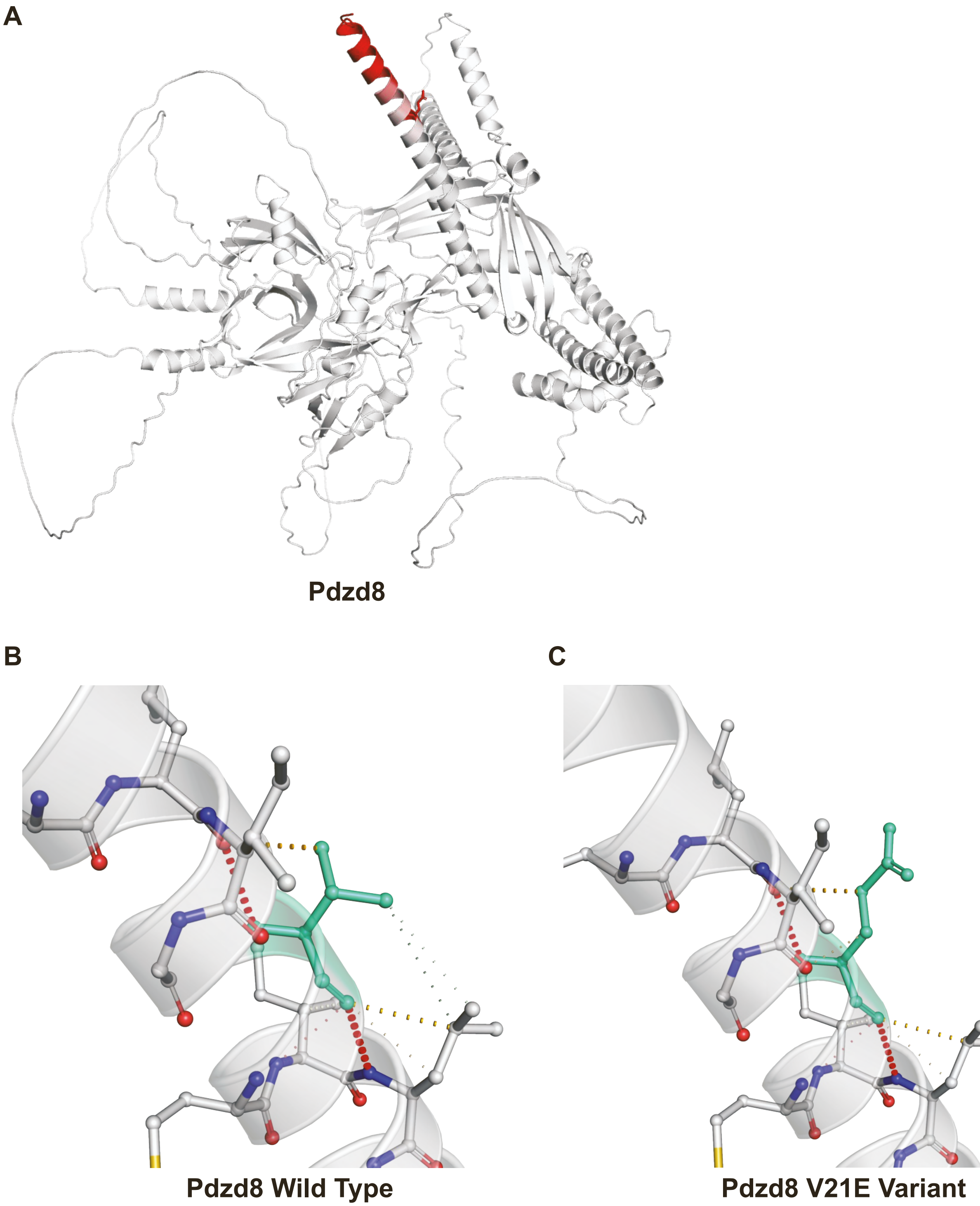

A- Predicted 3D structure of Pdzd8 using Dynamut. Red regions indicate residues predicted to undergo increased vibrational entropy (greater flexibility) upon mutation (corresponding to the N-terminal transmembrane domain). B- Close-up of the wild-type Pdzd8 transmembrane helix showing Val21 in green sticks with surrounding residues as gray sticks. Hydrogen bonds are shown as red dashed lines, hydrophobic contacts as yellow dashed lines, and weaker interatomic contacts as gray dashed lines.
C- Same view for the Pdzd8 V21E mutant, with Glu21 in green sticks and altered interatomic contacts shown as in (B). DynaMut predicted a modest stabilizing effect of the V21E mutation ($\Delta\Delta G$ = +0.071 kcal/mol), with mixed contributions across different predictors (EnCoM: –0.089 kcal/mol; mCSM: –0.087 kcal/mol; SDM: +1.480 kcal/mol; DUET: +0.662 kcal/mol). Vibrational entropy analysis suggested a slight increase in molecular flexibility ($\Delta\Delta S_{vib}$ = +0.111 kcal/(mol K)). DeepTMHMM analysis indicated no change in the position of the transmembrane helix (residues 8–31) (data not shown). Together, these results suggest that V21E does not alter transmembrane insertion but may subtly modulate local packing and flexibility.

Figure S6: Targeted sequencing confirms *Appl^d* specific alterations in CG3156 and Pdzd8.

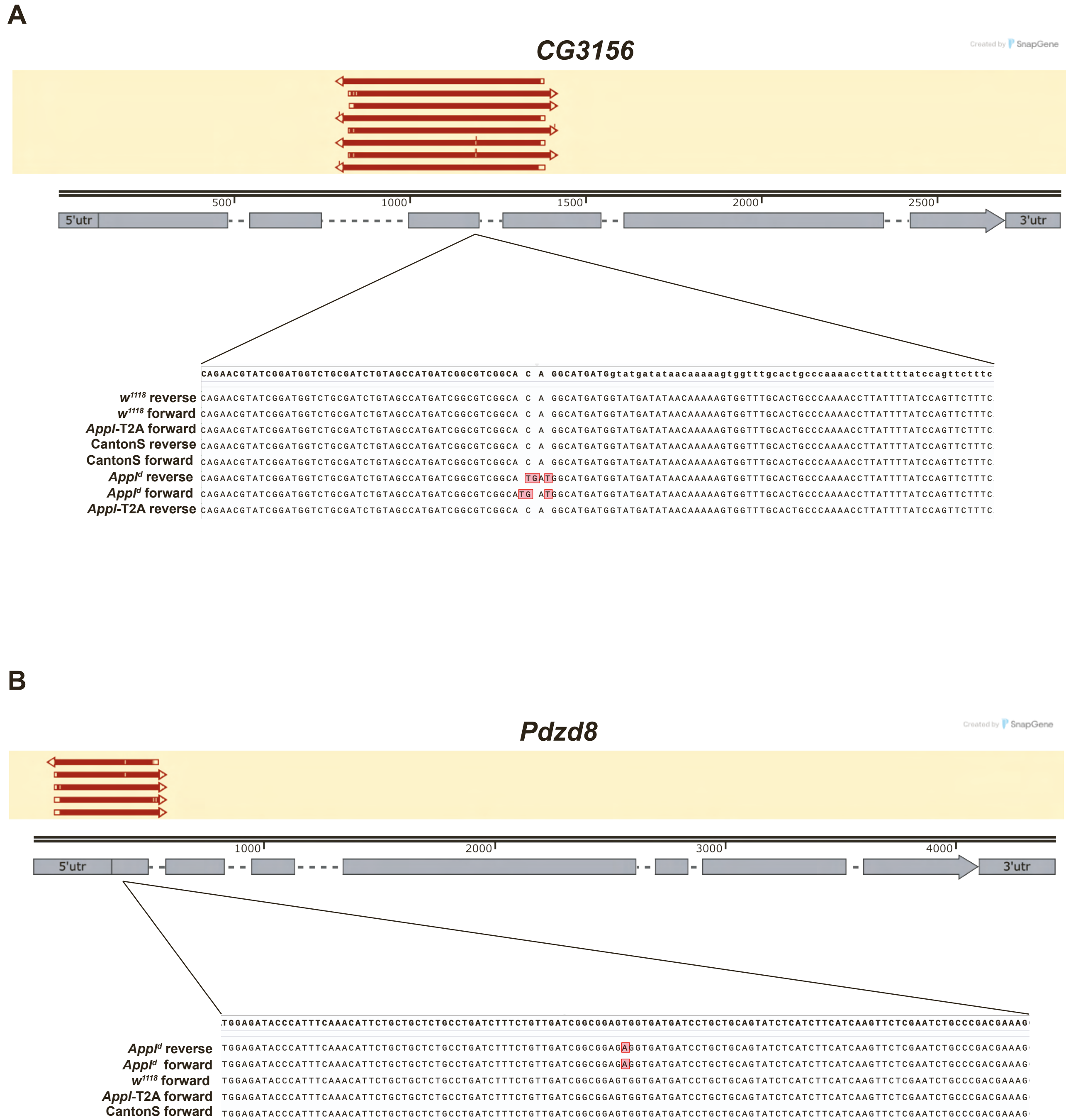

A/B- To confirm that the structural variants identified in our Appld assembly were unique to Appld, we performed targeted PCR and sequencing of the affected regions in *Appl^d*, *Appl*-T2A-GAL4, Canton-S, and *w^1118* flies. Sequence traces were aligned to the Drosophila reference genome (dm6) in SnapGene, which revealed that the lesions in CG3156 and Pdzd8 were present only in *Appl^d*, while all three control lines retained intact sequence. Panels show alignment of sequences to reference (top) and at the sequence level (bottom) for CG3156 (A) and Pdzd8 (B).

Figure S7: *Appl^d* proteomic changes show partial overlap between younger and older adults.

A Both Downregulated

Protein	Nithianandam Log(2)FC	Our Orbitrap Log(2)FC
Appl	-4.74	-6.64
Cyp6a13	-3.57	-6.64
Cyp309a1	-3.19	-6.64
Cyp6d2	-2.96	-6.64
Mocs2B	-1.67	-6.64
Slbp	-0.76	-6.64
Cpr49Ac	-0.53	-6.64
pyd	-0.41	-6.64
Cyp4p1	-3.46	-5.25
O-fut2	-0.39	-4.04
Amy-d	-0.38	-3.11
AttC	-2.47	-3.07
EG:171D11.2	-3.69	-2.89
Pepck2	-2.50	-2.02
Arc1	-2.11	-1.81
Cyp311a1	-1.27	-1.72
Crz	-1.18	-1.51
fon	-0.62	-1.50
CG5579	-0.73	-1.40
Gbp2	-0.89	-1.28
BcDNA:RH70154	-1.56	-1.27
NimB5	-0.58	-1.25
psh	-0.72	-1.23
CT15890	-0.43	-1.17
ND-MLRQ	-0.52	-1.15
Jheh3	-1.42	-1.05
AOX3	-3.64	-1.04
ine	-0.51	-0.99
SPH66	-0.51	-0.98
Gss2	-0.59	-0.96
BcDNA:AT13707	-0.76	-0.94
Acp76A	-2.99	-0.91
levy	-0.55	-0.90
Acer	-0.37	-0.80
mt:CoII	-0.37	-0.80
Ance-4	-0.34	-0.80
Ugt49B1	-1.44	-0.78
CREG	-0.82	-0.78
Alp4	-0.98	-0.75
rdgBbeta	-1.01	-0.75
COX4	-0.50	-0.75
MP1	-0.36	-0.75
Pld3	-0.33	-0.74
Acbp2	-0.50	-0.73
Abcd3	-0.85	-0.73
anon-EST:Posey66	-0.63	-0.73
c-SPH35	-0.49	-0.71
Lsd-2	-0.53	-0.71
l(2)34Fc	-0.44	-0.69
Ugt49C1	-1.58	-0.68
Jheh1	-0.55	-0.68
COX5A	-0.77	-0.66
cype	-0.54	-0.64
Obp99a	-0.63	-0.61
Lsd-1	-0.85	-0.58
Mal-A5	-0.65	-0.54
Nep7	-0.36	-0.51
Antdh	-0.99	-0.49
e	-1.03	-0.49

Both Upregulated

Protein	Nithianandam Log(2)FC	Our Orbitrap Log(2)FC
cysu	0.46	6.64
St1	0.50	6.64
Pkn	0.52	6.64
DIP-epsilon	1.10	6.64
Cpr65Av	1.16	6.64
Lsp1alpha	1.39	6.64
Cpn	4.83	6.64
BomBc1	5.30	6.64
dmCG15098	8.01	6.64
BomT3	17.50	6.64
BcDNA:AT09463	1.56	4.06
BcDNA.GH08385	1.31	2.52
St3	1.10	2.04
EG:BACR7A4.14	0.89	1.92
GstE14	0.56	1.54
GstE1	1.32	1.47
GstE8	0.77	1.44
Nemp	0.95	1.37
mle	0.84	1.05
Cyp6g1	2.68	0.99
Rpb7	0.43	0.93
Mal-B2	0.96	0.87
Had1	0.81	0.86
Cyp28d1	0.87	0.80
AGBE	0.38	0.79
CG12562	1.15	0.77
Su(P)	1.13	0.74
Ugt35A1	2.07	0.71
yellow-h	0.80	0.71
UK114	0.48	0.69
Rel	0.50	0.69
Lcp65Ac	0.79	0.67
Mgstl	0.64	0.66
Dcr-2	0.46	0.66
AsnS	0.63	0.66
Tsf1	0.68	0.64
Ugt35B1	4.96	0.64
Gart	0.83	0.55
Obp56d	0.82	0.52
Pu	1.04	0.52
smp-30	0.35	0.52
Dbp80	0.41	0.52
mEFTs	0.56	0.52
PPO2	0.46	0.50
Argl	1.25	0.49
GH26	0.47	0.47
GstD1	0.57	0.45
Pfas	1.15	0.45

Upregulated (Nithianandam)
Downregulated (this study)

Protein	Nithianandam Log(2)FC	Our Orbitrap Log(2)FC	Difference
CG15055	0.89	-6.64	7.53
NimB4	0.41	-6.64	7.06
Sin3A	0.41	-6.64	7.05
Cpr60D	0.36	-6.64	7.00
Polr2M	0.35	-6.64	6.99
Ire1	0.85	-2.57	3.41
Acp1	1.10	-2.17	3.27
BEST:GH10831	1.54	-1.20	2.74
Obp99b	1.51	-0.91	2.42
Vajk1	0.76	-1.56	2.32
Cpr57A	1.35	-0.85	2.20
Vajk3	0.85	-1.34	2.19
Lsp2	1.20	-0.84	2.04
Vajk2	0.86	-0.97	1.83
c-SPH64	0.45	-1.11	1.56
retinin	0.53	-0.92	1.44
CG3950	0.74	-0.69	1.44
dj-1beta	0.76	-0.50	1.27
Cpr97Ea	0.40	-0.83	1.23
Ugt37D1	0.48	-0.74	1.21
Cpr97Eb	0.38	-0.83	1.21
CG11955	0.36	-0.84	1.19
Yp2	0.51	-0.62	1.13

Downregulated (Nithianandam)
Upregulated (this study)

Protein	Nithianandam Log(2)FC	Our Orbitrap Log(2)FC	Difference
IM18	-4.22	6.64	10.86
Ku80	-3.61	6.64	10.26
SP	-3.29	6.64	9.93
S-Lap3	-2.97	6.64	9.62
S-Lap7	-2.88	6.64	9.53
lectin-29Ca	-2.78	6.64	9.42
Acp26Aa	-2.75	6.64	9.39
PGRP-SC2	-2.56	6.64	9.21
Acp32CD	-2.43	6.64	9.07
Spn77Bc	-2.35	6.64	9.00
CG17093	-2.28	6.38	8.66
PGRP-LB	-1.07	6.64	7.71
CG10220	-0.71	6.64	7.36
mRpL10	-0.43	6.64	7.07
Cyp9f3	-0.43	6.64	7.07
mRpL20	-0.36	6.64	7.01
cg9143	-0.32	6.64	6.97
Cyp4ac2	-2.64	0.83	3.47
DptB	-2.33	1.09	3.42
DptA	-2.38	1.02	3.40
FOHSDR	-0.45	2.16	2.62
Had2	-1.73	0.88	2.60
Mthfs	-1.09	0.95	2.04
cer	-1.14	0.58	1.71
Hsp22	-0.65	1.03	1.67
FBpp0074674	-0.41	1.14	1.54
TotC	-0.73	0.64	1.37
AGO2	-0.47	0.72	1.19

A- Heatmap comparisons of FC values from this study (on 3-5 day old) compared to previously published data on ~10 day old *Appl^d* flies. Data was filtered to only include proteins found in both data sets. Left panel shows proteins significantly downregulated in both datasets. Middle panel shows proteins upregulated in both datasets. Right top panel shows proteins downregulated in our study and upregulated in Nithianandam et al., 2023[26]. Right bottoms panel shows proteins upregulated in our study and downregulated in Nithianandam et al., 2023.

Figure S8: *Appl* mutant flies exhibit altered sleep architecture across light and dark phases.

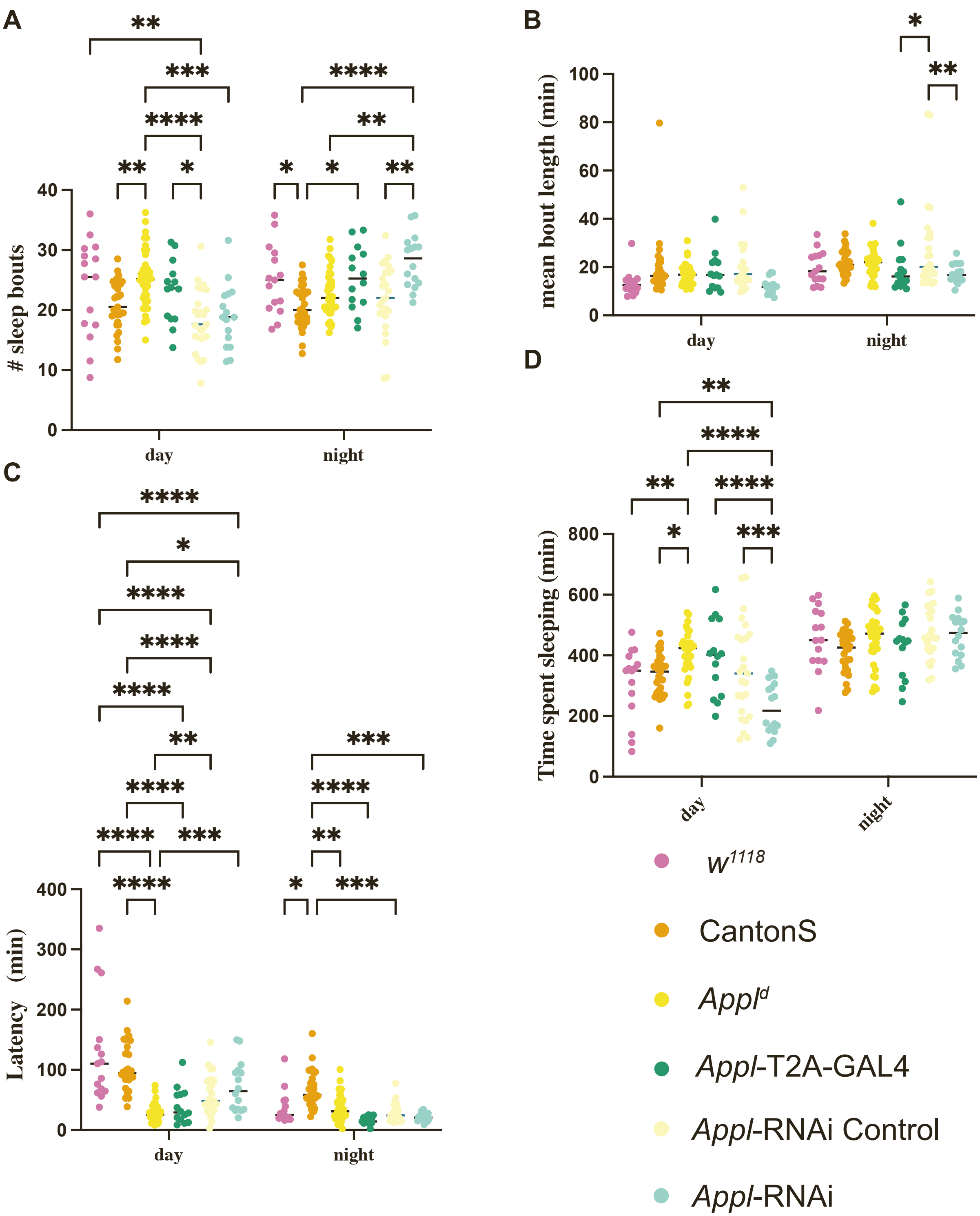

Sleep and activity profiles were assessed in adult male flies over consecutive days in a 12/12 light/dark cycle (LD) using the Drosophila Activity Monitoring System. Data were collected in 1-minute bins and analyzed using VANESSA-DAM. Sleep was defined as five or more consecutive minutes of inactivity. For all panels, daytime (light) metrics correspond to ZT0-12 and nighttime (dark) correspond to ZT12-24. A-D- Quantification of general sleep architecture during the day (left) and night (right). A- The number of sleep bouts per genotype. B- The mean duration of each sleep bout in minutes. C- Sleep latency, defined as the time in minutes before first sleep bout is initiated following light transition (ZT0 for day and ZT12 for night). D- The total time spent sleeping, measured in minutes. Significance was determined using two-way ANOVA followed by multiple comparisons; *p<0.05, **p<0.01, ***p<0.001, ****p<0.0001. Sample sizes the same as figure 7A-C.

Figure S9: Extended climbing analyses characterize genotype-dependent locomotor differences.

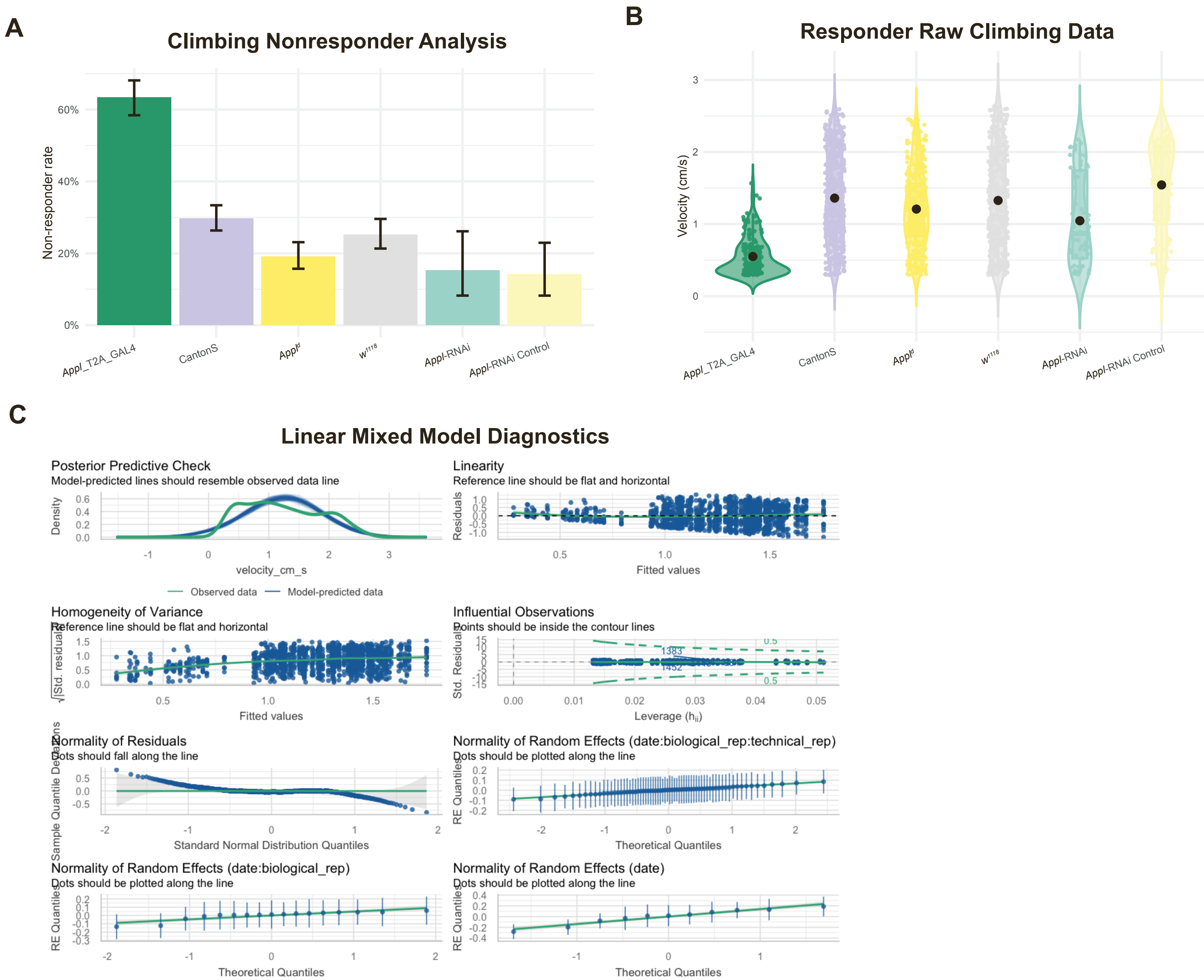

Additional data acquired during analysis of climbing behavior. A- Proportion of non-responding flies (velocity < 0.293 cm/s) calculated from the total pooled population for each genotype. Error bars represent 95% binomial confidence intervals. B- Raw distribution of climbing velocities for the responding population (velocity > 0.293 cm/s). These data served as the input for the Linear Mixed Model (LMM) presented in Figure 7. Individual points represent single flies in single experiments overlaid with violin and box plots to visualize data spread. C- Diagnostic plots confirming the assumptions of the LMM (Fixed effect: genotype; nested random effects: experimental date and cohort) were met. This panel includes a Residuals vs Fitted plot demonstrating homogeneity of variance across the range and a Quantile-Quantile plot confirming that the model residuals follow a normal distribution, thus validating the statistical framework.

Data S1: Original western blot images supporting immunoblot analysis.

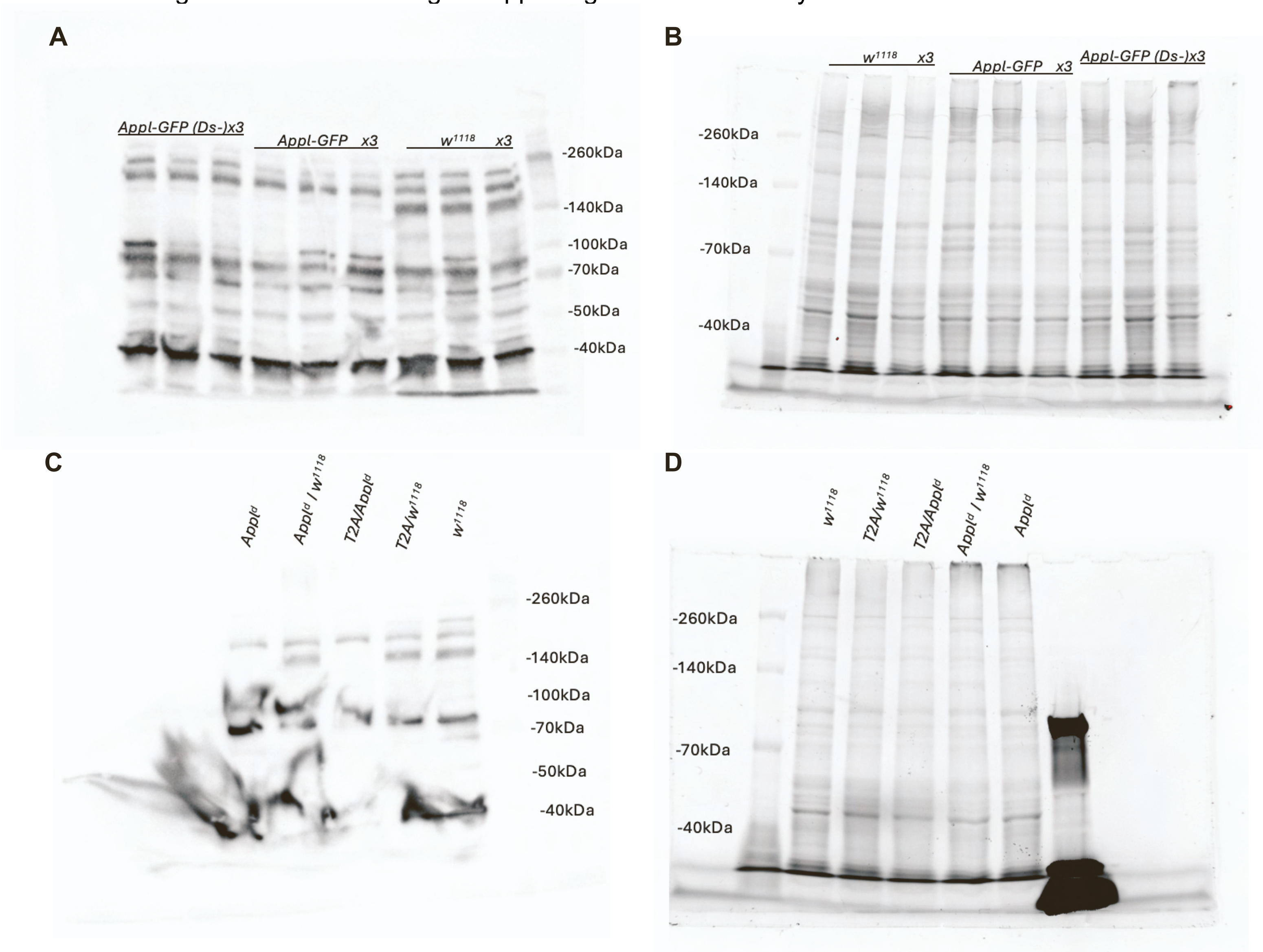

A- Raw anti-Appl western blot corresponding to Figure 4A. B- Corresponding stain-free gel image for the samples shown in panel A. C- Raw anti-Appl western blot corresponding to Figure 4B. D- Corresponding stain-free gel image for the samples shown in panel C. Molecular weight markers indicated on each image. Images are uncropped and shown without adjustment.
